# Carbon‐Based Flexible Electrode for Efficient Electrochemical Generation of Reactive Chlorine Species in Tumor Therapy

**DOI:** 10.1002/adhm.202500369

**Published:** 2025-05-24

**Authors:** Cuinan Jiang, Zhaoyu Chen, Ruihao Yang, Ziga Luogu, Qian Ren, Hao Hu, Kaixin Wang, Senlin Li, Changlin Deng, Meng Li, Lu Zheng

**Affiliations:** ^1^ Department of Hepatobiliary Surgery Xinqiao Hospital Third Military Medical University (Army Medical University) Chongqing 400038 China; ^2^ National Innovation Center for Industry‐Education Integration of Energy Storage Technology MOE Key Laboratory of Low‐Grade Energy Utilization Technologies and Systems CQU‐NUS Renewable Energy Materials & Devices Joint Laboratory School of Energy and Power Engineering Chongqing University Chongqing 400044 China

**Keywords:** carbon nanowire network, carbon vacancy, ferroptosis, flexible electrode, reactive chlorine species, tumor therapy

## Abstract

Reactive chlorine species (RCS) are alternatives to reactive oxygen species (ROS) in tumor therapeutics. Unlike ROS, whose generation is limited by hypoxic conditions or insufficient H_2_O_2_ levels in the tumor, RCS can be generated through the electrochemical oxidation of abundant Cl^−^ present in body fluids. However, traditional electrochemical therapy modalities have shown suboptimal outcomes. Herein, a flexible anodic electrode is fabricated by growing a carbon nanowire network (C‐NWN) onto carbon cloth (CC). Attributing to its excellent hydrophilicity, high specific surface area, and electrochemical surface area, CC@C‐NWN demonstrates a superior capability for RCS generation. Additionally, the carbon vacancies in CC@C‐NWN not only enhance Cl^−^ adsorption but also reduce the reaction free energy of the chlorine evolution reaction (CER) more significantly compared to that of the oxygen evolution reaction, thereby promoting the CER process. RCS generated from the CC@C‐NWN electrochemical system induces severe oxidative stress, disrupting the redox homeostasis in tumor cells and promoting the synergistic anti‐tumor effect of apoptosis and ferroptosis. The pliability of CC@C‐NWN enables it to conform closely to the tumor, and it has demonstrated remarkable tumor‐suppressive efficacy under low‐voltage (3 V) condition in in vivo experiments. Therefore, the work holds significant promise for the development of novel tumor treatment strategies.

## Graphical abstract

A flexible electrode loaded with a carbon nanowire network (CC@C‐NWN) is developed for electrochemically generating reactive chlorine species (RCS) from abundant chloride ions in body fluids for tumor therapy. CC@C‐NWN enhances chlorine evolution, inducing redox dysregulation and synergistic apoptosis‐ferroptosis in tumor cells. Remarkable tumor suppression is demonstrated under low‐voltage conditions in vivo, highlighting its clinical potential for RCS‐based antitumor therapy.

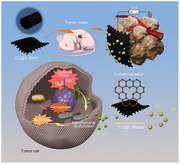

## 1 Introduction

Cancer cells typically exhibit a relatively severe state of oxidative stress.^[^ ^1^, ^2^, ^3^, ^4^ ^]^ This condition makes them more susceptible to damage induced by oxidative species compared to normal cells.^[^ ^5^, ^6^, ^7^ ^]^ Reactive oxygen species (ROS) directly inflict oxidative damage to the components of cancer cells.^[^ ^8^, ^9^, ^10^ ^]^ Therefore, ROS can effectively combat various types of tumors without inducing resistance.^[^ ^11^, ^12^, ^13^ ^]^ Motivated by this biochemical property, numerous tumor treatment modalities that are capable of generating ROS have been developed, including radiotherapy,^[^ ^14^ ^]^ photodynamic therapy,^[^ ^15^ ^]^ and chemodynamic therapy.^[^ ^16^ ^]^ However, the synthesis of ROS largely depends on the concentrations of intracellular O₂ or H₂O₂.^[^ ^17^, ^18^ ^]^ Consequently, the hypoxic microenvironment and insufficient endogenous H₂O₂ levels in tumors restrict the therapeutic efficacy of these ROS‐based treatments.^[^ ^19^, ^20^, ^21^, ^22^, ^23^, ^24^, ^25^ ^]^

By applying direct current (DC), the electrolysis of chloride salt solutions such as NaCl, can generate reactive chlorine species (RCS), including HOCl and ClO^−^.^[^ ^26^ ^]^ This process is referred to as the electrochemical chlorine evolution reaction (CER). Considering that Cl^−^ is the most abundant anion in human body fluids, applying the CER principle to tumor treatment can provide sufficient substrates for the electrochemical exogenous synthesis of RCS. Thereby, this strategy overcomes the obstacles posed by hypoxic tumor microenvironments or insufficient H_2_O_2_ levels. RCS exhibit significant antitumor potential. As a key active component of RCS, HOCl belongs to the non‐radical category of ROS and demonstrates potent oxidative capacity, as evidenced by its high oxidation potential (+1.49 V). The rate constant of the reaction between HOCl and reduced glutathione (GSH) is 3 × 10^7^ M^−1^S^−1^, which is significantly higher than that of other types of non‐radical ROS.^[^ ^18^ ^]^ HOCl exhibits strong antimicrobial activity against various types of microorganisms and demonstrates a significant cytotoxic effect in tumor cells.^[^ ^27^, ^28^ ^]^ Studies have shown that the attack of tumor cells by neutrophils is mediated by HOCl synthesized through the peroxidase/ H₂O₂/ halide system.^[^ ^29^, ^30^, ^31^ ^]^ One of the therapeutic mechanisms of traditional electrochemical therapy (EChT) also involves this characteristic of electrochemical CER.^[^ ^32^, ^33^ ^]^ However, due to the limited catalytic surface area of the electrode needles, it is inadequate to produce sufficient RCS. For large or irregularly shaped tumors, multiple electrode arrays are often needed,^[^ ^34^, ^35^ ^]^ which not only increases costs but also exacerbates the patient's discomfort.^[^ ^36^ ^]^ More serious is the fact that multiple electrode needle channels may increase the risk of local metastasis.^[^ ^37^, ^38^ ^]^ Research findings of generating chlorine radicals (·Cl) via near‐infrared (NIR) light for antitumor therapy is encouraging, as it effectively avoided the obstacle posed by tumor hypoxic condition.^[^ ^39^ ^]^ However, NIR light can be attenuated by tissue, thereby reducing the therapeutic efficacy.^[^ ^40^, ^41^ ^]^ Recent advancements in electrodynamic therapy combined with nanoparticles have shown promising results.^[^ ^42^ ^]^ However, the application of high voltage (20 V) may increase patient discomfort, as 10 V is generally considered the upper limit of tolerable voltage for conscious patients.^[^ ^35^ ^]^ Anodes fabricated from elements such as Ir and Ru, which are known for their superior CER and oxygen evolution reaction (OER) performance and excellent stability, are considered state‐of‐the‐art anode catalytic materials for CER.^[^ ^43^, ^44^ ^]^ However, elements Ir and Ru are rare and extremely scarce in Earth crust, making their large‐scale application challenging to sustain.^[^ ^45^, ^46^ ^]^ Additionally, studies have revealed that during the electrolysis process at the anode, the majority of the energy were consumed in the OER process.^[^ ^47^, ^48^ ^]^

Therefore, designing an anode catalyst electrode that is economical, readily available, biocompatible, and capable of efficiently producing RCS under low‐voltage conditions is of great significance for enhancing the therapeutic effect of RCS‐based tumor treatment. Carbon‐based materials, owing to their superior chemical, electronic, and catalytic properties, have consistently been at the forefront of chemical and materials science research. They possess advantages such as high efficiency, low cost, and environmental cleanliness.^[^ ^49^, ^50^, ^51^ ^]^ Among then, carbon cloth (CC), as a flexible conductive material, exhibits marvelous mechanical strength, electrical conductivity, and corrosion resistance.^[^ ^52^ ^]^ It can function as a conductive substrate for biosensors and provides suitable conditions for the development of cell‐derived matrix proteins, thereby demonstrating excellent biocompatibility.^[^ ^53^, ^54^, ^55^ ^]^ More importantly, human tissues are more compatible with this kind of flexible material. In addition, the soft nature of CC enables it to conform well to the complex shapes and sizes of tumors. Therefore, CC represents an exceptionally optimal substrate material for bioelectrodes.

**Scheme 1:**
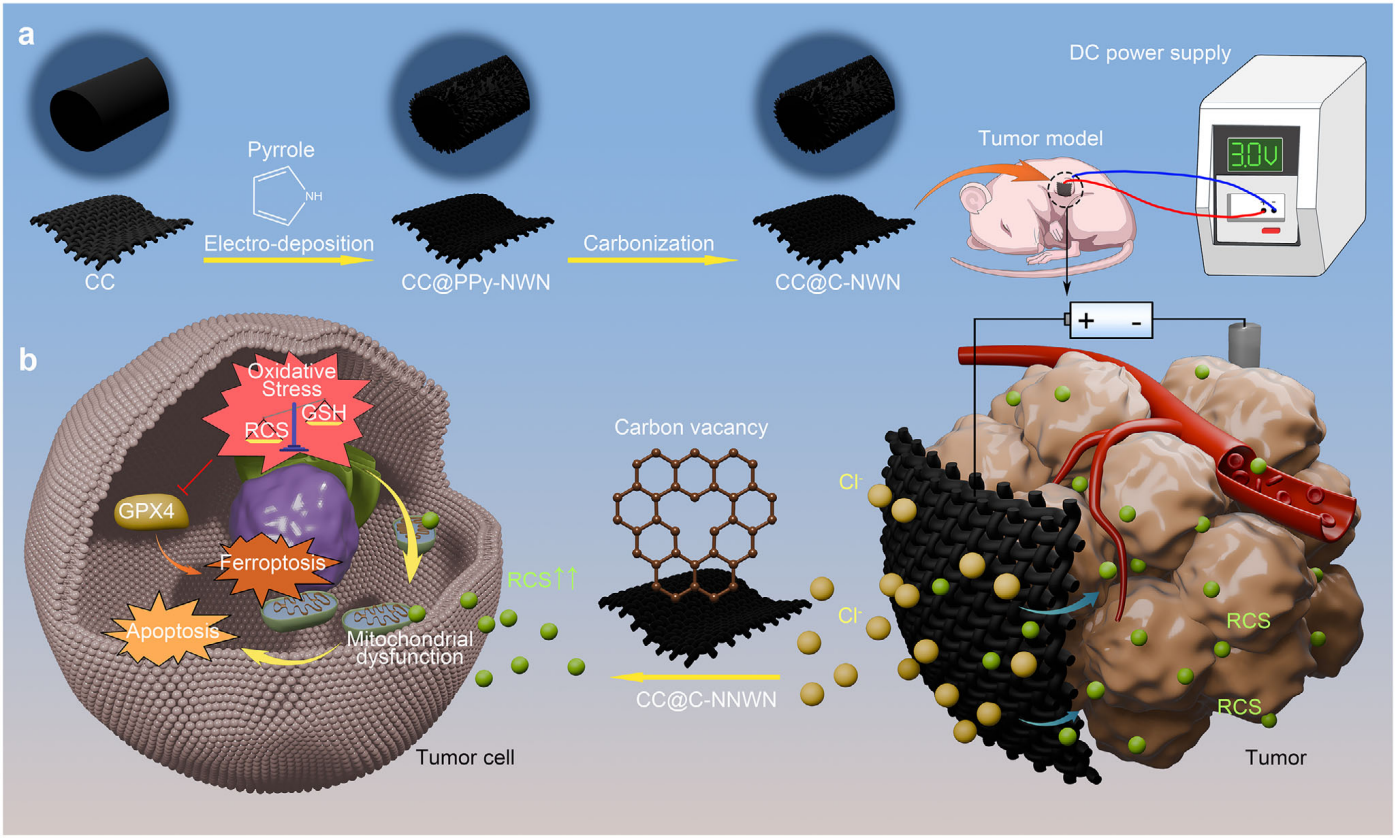
Carbon cloth @ Carbon‐nanowire network (CC@C‐NWN) for reactive chloride species (RCS)‐based tumor therapy. a) The fabrication process of CC@C‐NWN. Implanting CC@C‐NWN as the anode into the tumor effectively suppresses tumor growth under a 3 V direct current (DC) voltage drive. b) With its higher specific surface area and electrochemical surface area, as well as the presence of carbon vacancies, CC@C‐NWN significantly enhances the generation of RCS. RCS disrupts the redox homeostasis in tumor cells, thereby inducing severe oxidative stress, mitochondrial dysfunction and ultimately promoting the synergistic anti‐tumor effect of apoptosis and ferroptosis.

Herein, we designed a carbon‐based flexible catalytic anode electrode capable of efficiently catalyzing the generation of RCS for tumor treatment (**Scheme** **1**). The electrode was fabricated using CC as the substrate, followed by the in situ growth of carbon nanowire network (C‐NWN) onto the CC fibers (CC@C‐NWN). The soft nature of CC@C‐NWN enabled flexible cutting according to the tumor size and ensure conformal adhesion to its shape, thereby effectively increasing the therapeutic contact area with the tumor. Owing to the exceptional electrical conductivity of the carbon material and the extensive specific surface area provided by the nanowire network, CC@C‐NWN exhibited more efficient RCS production under 3 V DC conditions compared to CC and platinum (Pt) electrodes. Additionally, CC@C‐NWN exhibited superior hydrophilicity, which improve the efficiency of ion conduction and the electrode utilization rate. Moreover, we revealed that the carbon nanowire are rich in unsaturated oxygen atoms and carbon vacancy, which facilitate the adsorption of Cl^−^ and enhance the charge polarization effect, thereby significantly benefit the electrocatalytic performance.^[^ ^56^, ^57^ ^]^ Meanwhile, the theoretical calculations indicated that the theoretical overpotential for the CER (ηCER) of CC@C‐NWN is reduced more significantly compared to that for the OER (ηOER), thereby making the CER process more favorable over OER. In electrochemical performance tests, CC@C‐NWN demonstrated a lower CER overpotential, a smaller Tafel slope and a larger electrochemical surface area (ECSA). The CC@C‐NWN electrochemical system induced intense oxidative stress and disrupted the redox homeostasis of tumor cells, resulting in a synergistic anti‐tumor effect via apoptosis and ferroptosis. The in vivo experiments confirmed that CC@C‐NWN exhibits excellent biocompatibility and can effectively inhibit tumor growth under low‐voltage (3 V) and a short treatment time. Our work provides a valuable reference for the future development of economically viable, environmentally friendly, and convenient RCS‐based tumor treatment modalities.

## 2 Results and Discussion

### 2.1 Characteristics of CC@C‐NWN

**Figure 1:**
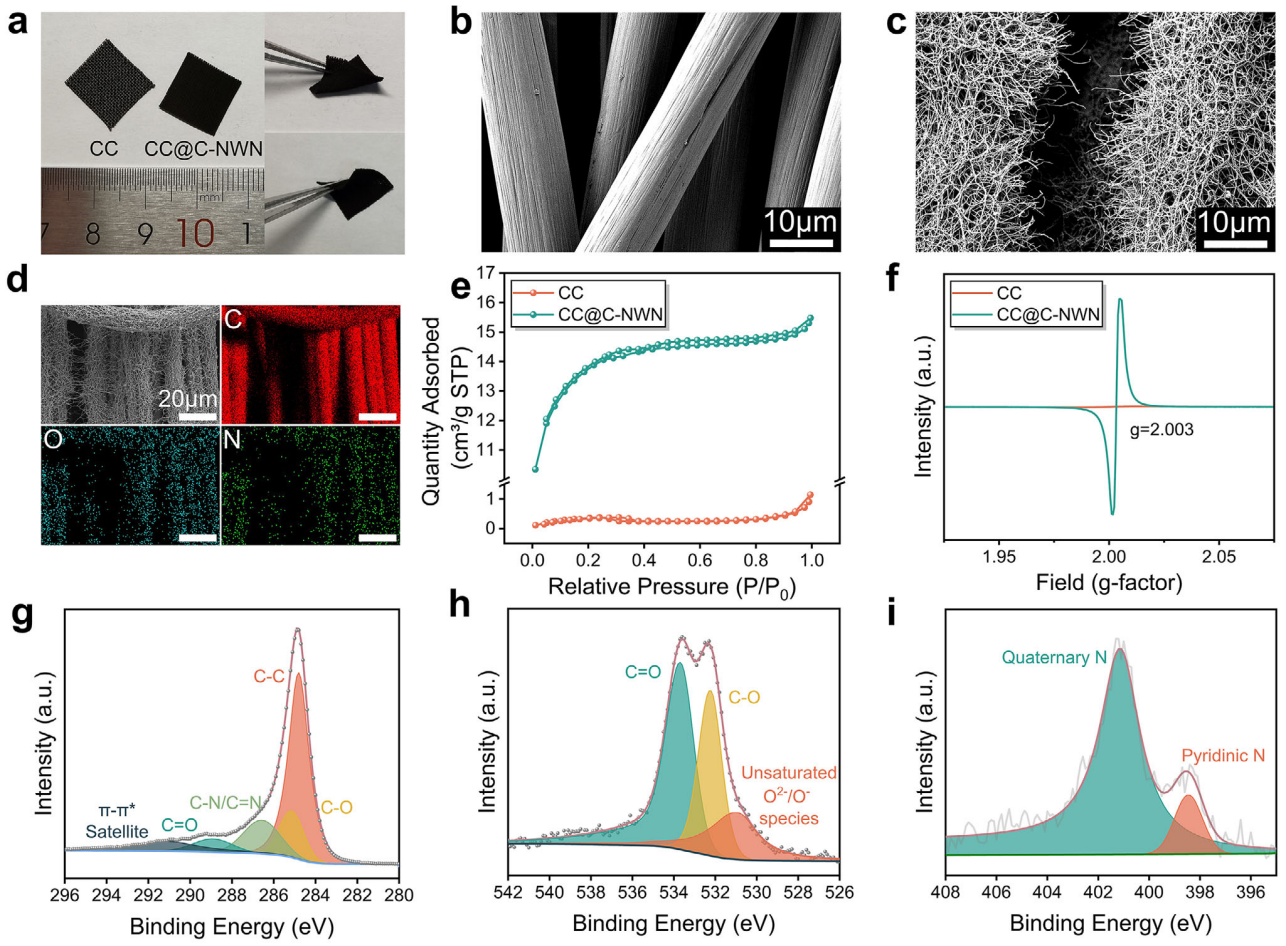
a) The appearance of CC and CC@C‐NWN. b) SEM image of bare CC fibers. c) SEM image of CC@C‐NWN. d) EDS elemental mapping of C, O, and N in CC@C‐NWN. e) N_2_ adsorption‐desorption isotherm of CC and CC@C‐NWN. f) EPR spectra of CC and CC@C‐NWN. XPS spectra of C1s (g), O1s (h) and N1s (i) of CC@C‐NWN.

From the appearance of the naked eye, the color of CC@C‐NWN became darker than that of CC. CC@C‐NWN maintained its flexibility and hydrophilic properties (**Figure** **1**a; Figure S1, Supporting Information). The flexibility of CC@C‐NWN enabled it to be precisely tailored to fit the dimension of the tumor, ensuring conformal adhesion to its morphology. This significantly enhanced the therapeutic contact area with the tumor. The superior hydrophilicity of the material enhanced the ionic conductivity and the utilization rate of the electrode. Scanning electron microscopy (SEM) confirmed the successful growing of C‐NWN (Figure 1c) on the bare CC fibers (Figure 1b), and its nanostructure was well inherited from that of the pre‐carbonized precursor polypyrrole nanowire network (PPy‐NWN) (Figure S2, Supporting Information). The diameter of the C‐NWN was ≈50–200 nm (Figure S3, Supporting Information). The energy‐dispersive X‐ray spectroscopy (EDS) exhibited the homogeneous distribution of C, O and N elements on the surface of the CC@C‐NWN (Figure 1d; Figure S4, Supporting Information). In Brunauer Emmett Teller (BET) experiment, the N_2_ adsorption isotherm of CC@C‐NWN exhibited a sharp uptake at low relative pressures (P/P0), without any significant hysteresis, indicating the reversibility of monolayer adsorption. At medium and high relative pressures, a hysteresis effect was observed, with the appearance of a hysteresis loop, which was characteristic of H4‐type loops (Figure 1e). This suggested the presence of both micropores and mesopores within the material. The pore size distribution (PSD) of CC@C‐NWN also revealed the coexistence of micropores and mesopores (Figure S5, Supporting Information). A distinct peak in the PSD was observed at 1.74 nm, indicating microporous characteristics. Furthermore, an additional PSD peak was discernible, centered at 3.4 nm, suggesting the presence of mesoporous structures within the material. The specific surface areas of CC and CC@C‐NWN were calculated to be 1.4 and 50.6 m^2^/g, respectively. This confirmed that the growth of C‐NWN onto CC resulted in a substantial increase in specific surface area, consequently offering an expanded array of reactive sites for the CER. In the X‐ray diffraction (XRD) pattern of CC@C‐NWN, the peaks at ≈25° and 44° corresponded to the (002) and (101) crystal planes of graphite, indicating that the PPy‐nanowire has been fully transformed into carbon nanowire (Figure S6, Supporting Information). The electron paramagnetic resonance (EPR) spectrum of CC@C‐NWN exhibited a strong signal peak at g ≈ 2.003 (Figure 1f). The strong EPR signal observed at g ≈ 2.003 in carbon materials is a characteristic of vacancy‐type defects, attributed to the unpaired electrons generated by sp^2^‐hybridized carbon atoms in π‐conjugated aromatic rings.^[^ ^58^, ^59^ ^]^ Carbon defects, due to their unsaturated coordination, typically exhibited charge polarization effects, which were highly beneficial for electrocatalytic performance.^[^ ^57^, ^60^ ^]^ The X‐ray photoelectron spectroscopy (XPS) survey spectra of CC@C‐NWN further illustrated the coexistence of C, O and N elements (Figure S7, Supporting Information). The proportion of C, O, and N in CC@C‐NWN was 85.23%, 12.06%, and 2.71% respectively. The high resolution C1s XPS spectrum showed a dominant peak at 284.8 eV and four small peaks at 285.2, 286.6, 289, and 291.2 eV (Figure 1g). The peak at 284.8 eV was attributed to C─C bonding, signal at 285.2 eV corresponded to the C─O bonding, the peak centered at 286.6 eV was attributed to C─N/C═N bonding, and the peak at 289 eV was assigned to the C═O bonding,^[^ ^61^, ^62^, ^63^ ^]^ indicating that the C atom in CC@C‐NWN exist in different chemical states. A π–π* satellite peak was observed at 291.2 eV, also referred “plasmon loss” or “shake‐up,” specifically originated from sp^2^‐hybridized carbon.^[^ ^64^ ^]^ The π–π* satellite peak has also been observed in some highly conductive carbon materials, as well as in thermally reduced graphene oxide.^[^ ^65^, ^66^ ^]^ The emerge of this peak might be attributed to the changes of the electronic environment of the carbon material.^[^ ^67^, ^68^ ^]^ The O1s XPS spectrum of CC@C‐NWN could be fitted into three peaks (Figure 1h). The peak centered at 532.2 eV was attributed to the C─O bond, while the peak with a binding energy of 533.7 eV corresponded to the C═O bond. The peak with a binding energy of 531 eV corresponded to low oxygen coordination from unsaturated O^2−^/O^−^ species.^[^ ^69^, ^70^ ^]^ This suggested that unsaturated O^2−^/O^−^ groups are anchored on carbon defects, and the oxygen atoms possess unpaired electrons or unoccupied orbitals, thus benefiting the adsorption of reactants.^[^ ^71^ ^]^ However, this peak was not detected in the O1s spectrum of the CC (Figure S8, Supporting Information). In the N1s spectrum of CC@PPy‐NWN, the main peak at 400.1 eV corresponded to pyrrolic nitrogen (Figure S9, Supporting Information), whereas in the N1s spectrum of CC@C‐NWN, the main peak shifted to 401.1 eV (Figure 1i), representing the quaternary N. This indicated that the CC@C‐NWN sample has been substantially graphitized, and most of the nitrogen atoms doped into the graphitic‐like framework.^[^ ^72^ ^]^ Additionally, a minor peak at 398.4 eV in the N1s spectrum of CC@C‐NWN could be observed, corresponding to pyridinic N.^[^ ^72^ ^]^

### 2.2 Electrochemical Characteristics and CER Performance

**Figure 2:**
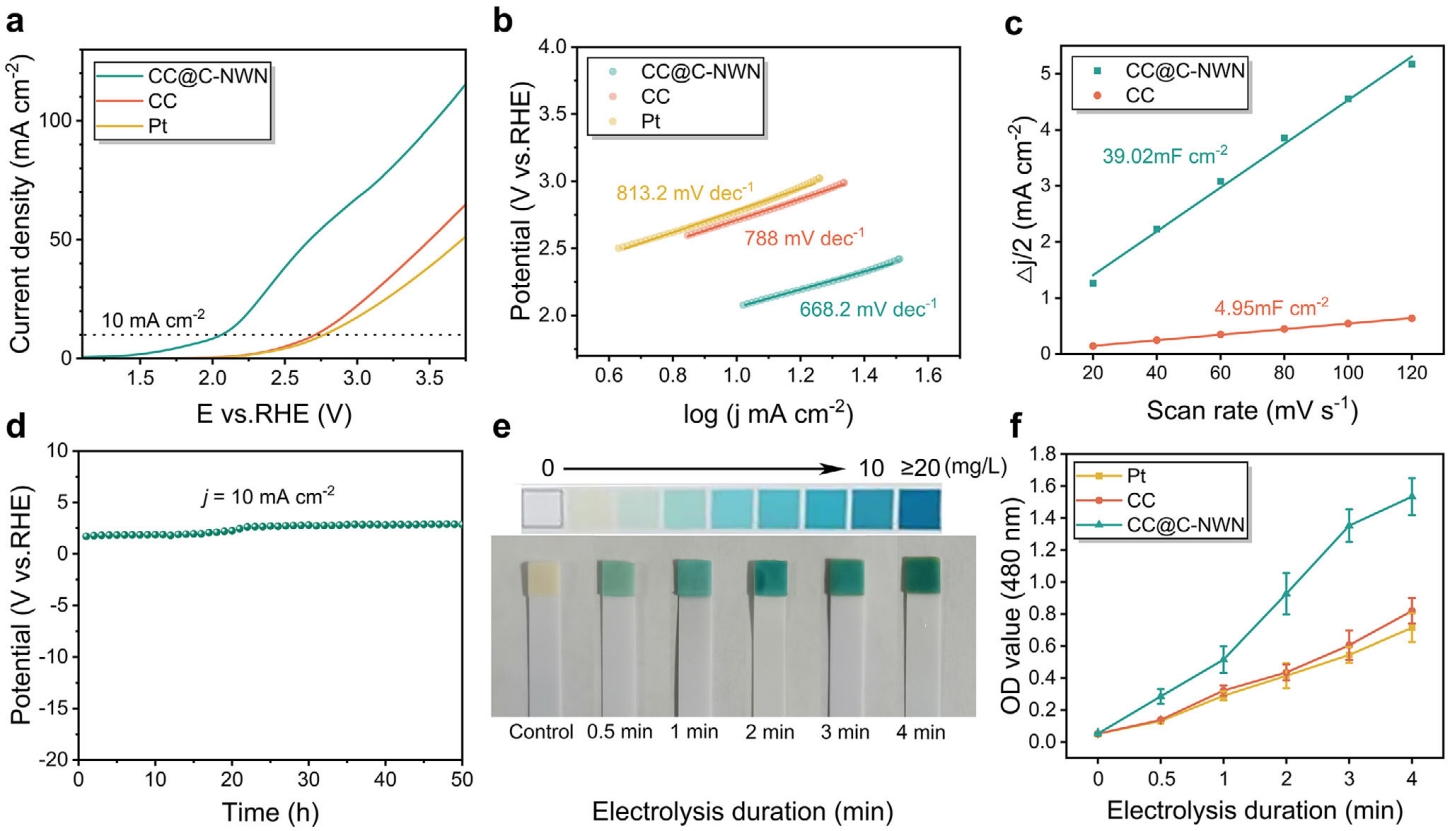
a) LSV curves in SBF (pH 7.2–7.4). b) Tafel plots of Pt, CC and CC@C‐NWN. c) Calculated C_dl_ values for CC@C‐NWN and CC. d) Chronopotentiometric testing curve measured at the current density of 10 mA cm^−2^ for CC@C‐NWN. e) Color changes of the residual chlorine test strip for CC@C‐NWN electrochemical system. f) Absorbance changed over electrolysis duration for the Pt, CC, and CC@C‐NWN electrochemical systems following the addition of DPD.

The electrochemical performance for CER of CC@C‐NWN was evaluated in simulated body fluid (SBF), which mimics the ionic composition and concentration of human blood plasma (Table S1, Supporting Information), maintaining an ion concentration analogous to that found in human bodily fluids. According to the linear sweep voltammogram (LSV) curves, the CC@C‐NWN electrode provided a current density of 10 mA cm^−2^ at an overpotential of 702 mV versus RHE, which is lower than that of commercial CC (1.36 V vs RHE) and Pt electrode sheet (1.41 V vs RHE) (**Figure** **2**a). This indicated that the CC@C‐NWN sample exhibits the highest catalytic activity for CER. The corresponding Tafel plots revealed that the CC@C‐NWN sample exhibits a smaller Tafel slope of 668.2 mV dec^−1^ in the potential range of 2.0 to 3.0 V versus RHE, compared to the CC (788 mV dec^−1^) and Pt electrode (813.2 mV dec^−1^) (Figure 2b). This indicated that the CC@C‐NWN sample possesses the fastest kinetics for the CER. Subsequently, the electrochemical double‐layer capacitance (C_dl_) of the CC and CC@C‐NWN catalytic electrodes were investigated using cyclic voltammetry (CV) at various potential scan rates to evaluate their ECSA (Figure S10, Supporting Information). The calculated C_dl_ values for CC@C‐NWN and CC were 39.02 and 4.95 mF cm^−2^, respectively (Figure 2c). This indicated that the CC@C‐NWN electrode possess the highest ECSA. To further investigate the effect of surface structure on catalytic activity, COMSOL simulations were performed to study the mass transfer behavior of Cl⁻ in the electrode material (The simulation details are shown in Tables S2–S4, Figure S11, Supporting Information). As shown in Figure S11 (Supporting Information), 2D geometric model of CC and C@CC‐NWN fiber were set up. The comparisons between planar electrodes and nanofibrous structured electrodes revealed that under identical electrochemical conditions, nanostructure promote local Cl⁻ enrichment (Figure S12, Supporting Information), significantly enhancing the Cl⁻ concentration gradient at the electrode‐electrolyte interface compared to planar structures and facilitating the CER reaction. This result further demonstrated the advantages of nanostructured electrodes. The chronopotentiometric curve demonstrated that the CC@C‐NWN electrode exhibit excellent stability at a current density of 10 mA cm^−2^ after 50 h of chronopotentiometric test (Figure 2d). After 50 h of continuous electrolysis, the nanowire network structure was well preserved, confirming that CC@C‐NWN has good structural stability (Figure S13, Supporting Information). In addition, there was no significant change in the elemental composition (Figure S14, Supporting Information).

Subsequently, we employed CC@C‐NWN (1.5 × 1.5 cm) as the anode, platinum plate electrode served as the cathode, to qualitatively and quantitatively evaluate the RCS generation performance of Pt, CC and CC@C‐NWN electrode under the 3 V DC condition, and compared the RCS generation efficacy between the three electrodes. As the electrolysis duration increased, the residual chlorine test strips exhibited a progressively deeper blue color (Figure 2e). Utilizing the color reaction principle between N, N‐diethyl‐p‐phenylenediamine (DPD) and RCS,^[^ ^73^, ^74^ ^]^ 130 mg DPD was introduced into the electrochemical system to induce color reaction, and the absorbance at the absorption wavelength of 480 nm was analyzed. The results showed that with the electrolysis duration increase, the red color of the electrochemical reaction system gradually deepen, and the absorbance correspondingly increased (Figure 2f; Figure S15, Supporting Information). At all the electrolysis time points, the CC@C‐NWN exhibited the highest absorbance, suggesting the most effective catalytic performance of RCS generation. This outcome supported the results of electrochemical performance testing.

### 2.3 DFT Calculation

According to the EPR and XPS results, the surface of CC@C‐NWN was rich in carbon vacancies and unsaturated oxygen. In order to further explore the main factors contributing to the enhancement of catalytic activity, DFT calculations were carried out to study the influence of carbon vacancies and unsaturated oxygen on the surface chemical environment. As shown in Figure S16 (Supporting Information), the pure carbon model (C), carbon vacancy model (Cv), and unsaturated oxygen model (Co) were constructed. As shown in Figure S17a (Supporting Information), the results indicated that the adsorption energy of Cl⁻ on the Cv surface (Eads (Cv) = ‐2.31 eV) is significantly stronger than those on the oxygen‐modified surfaces (Eads (Co) = −1.77, 0.32, 0.45, and 0.73 eV, respectively), unequivocally demonstrating the dominant contribution of carbon defects to Cl⁻ adsorption. Furthermore, density of states (DOS) calculations revealed that carbon vacancies induce significant electronic states near the Fermi level, a feature lacking in oxygen‐functionalized surfaces, thereby facilitating enhanced electron transfer during Cl⁻ binding (Figure S17b, Supporting Information). Additionally, the work function of the carbon‐defect surface (4.259 eV) was markedly lower than that of oxygenated surfaces (4.407 eV, as illustrated in Figure S18, Supporting Information), directly confirming that defect engineering amplifies surface charge polarization by promoting electron delocalization. These results collectively demonstrated that carbon defects are the primary drivers of both Cl⁻ adsorption enhancement and charge polarization effects, whereas unsaturated oxygen atoms play a secondary role in fine‐tuning the local coordination environment.

**Figure 3:**
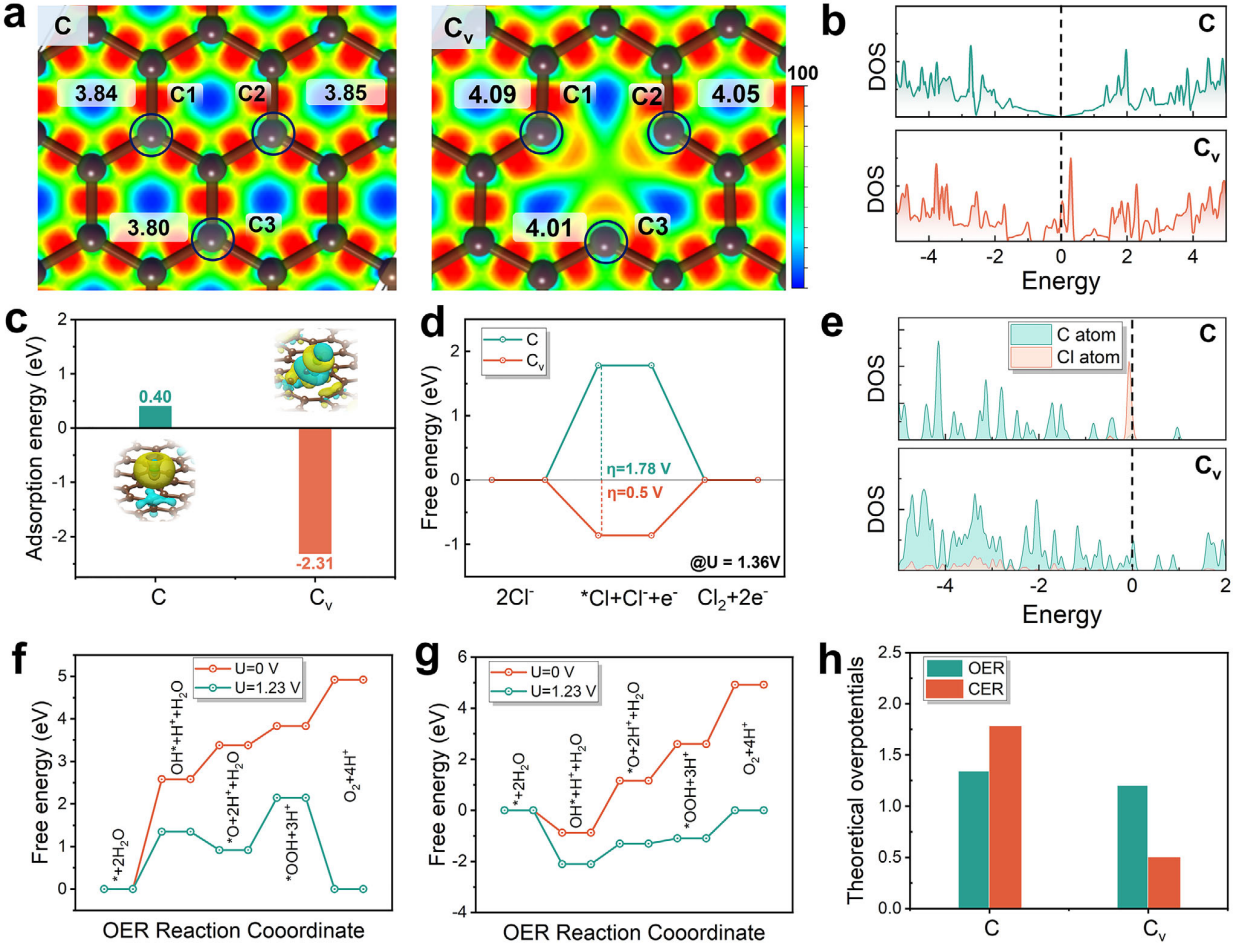
a) ELF plot of C and Cv models. b) DOS of C and Cv models. c) The Cl^−^ ion adsorption energy. Insert: CDD plot in Cl^−^. Isovalue is 0.002 eV. d) Free energy diagrams of CER on all C and Cv models. e) DOS of C and Cl atom of C and Cv model. The free energy diagrams of OER over f) C and g) Cv models. h) The OER and CER theoretical overpotential for C and Cv model.

To further clarify the effect of carbon vacancies on CER activity and the mechanism of CC@C‐NWN, the electron localization function (ELF) plots were studied. As shown in **Figure** **3**a, the ELF plots revealed a higher delocalization of electrons on the exposed edge carbon atoms. The Bader charge suggested that the introduction of carbon vacancies increase the electronic charge of the edge carbon atoms (C1, C2, and C3). Owing to the unique bonding environment of these edge atoms, the resulting local charge redistribution enriched the charge density at these sites, thereby enhancing their potential as electrocatalytic active sites.^[^ ^75^ ^]^ The DOS results suggested that, compared to the C model, the Cv model exhibits electronic states near the Fermi energy level, which may improve the conductivity of CC@C‐NWN (Figure 3b).

To gain an in‐depth investigation into the effect of carbon vacancy introduction on the CER mechanism, the adsorption energy of *Cl and *OCl intermediates on C and Cv models were considered in this work. As shown in Figure 3c and Table S5 (Supporting Information), the adsorption energy calculations suggested that the adsorption of *Cl is more favorable in both the C and Cv models.^[^ ^76^ ^]^ Thus, the CER mechanism was likely to proceed via the Volmer–Heyrovsky (V–H) pathway. Figure 3d depicted the Gibbs free energy change of CER with respect to the C and Cv models at an equilibrium potential of 1.36 V via the V–H pathway. The results indicated that, upon the introduction of carbon vacancies, the rate‐determining step of the CER reaction shifts from the adsorption of Cl⁻ to the desorption of Cl⁻, owing to the stronger adsorption of Cl⁻ by Cv. The η_CER_ for the C and Cv models were 1.78 V and 0.5 V, respectively. The lower theoretical overpotential for Cv suggested that carbon vacancies facilitate enhanced CER performance. The DOS plots for C and Cl in both the C and Cv models revealed that in the C model, the DOS of Cl atom shows strong localization, whereas in the Cv model, the Cl atom diffuses more freely (Figure 3e). This further demonstrated that carbon vacancies improve the adsorption of Cl⁻ by the catalyst. To better understand the high selectivity for Cl₂, the reaction free‐energy diagrams for the OER and CER were compared in this study. As shown in Figure 3f, for the C model, the η_OER_ was 1.35 V. On the Cv model, the η_OER_ reduced to 1.21 V (Figure 3g). Combined with the above results, for the C model, the η_OER_ was lower than the η_CER_ (1.78 V), rendering the OER more favorable. However, for the Cv model, the introduction of carbon vacancies significantly reduced the η_CER_, leading to η_CER_ < η_OER_, and enhanced the Cl₂ selectivity in CC@C‐NWN (Figure 3h).^[^ ^77^, ^78^ ^]^

Overall, DFT calculations demonstrated that the introduction of carbon vacancies increases the active sites and enhances the catalyst activity of the electrode. The Cl^−^ catalyzing reaction of Cv primarily proceeded via the V–H pathway, due to the strong interaction of the edge carbon atoms with Cl^−^, the theoretical overpotential reduced, thus favoring the process of CER reaction. In addition, the introduction of carbon vacancies significantly improved Cl_2_ selectivity compared to that of pure carbon.

### 2.4 In Vitro Antitumor Performance of CC@C‐NWN

**Figure 4:**
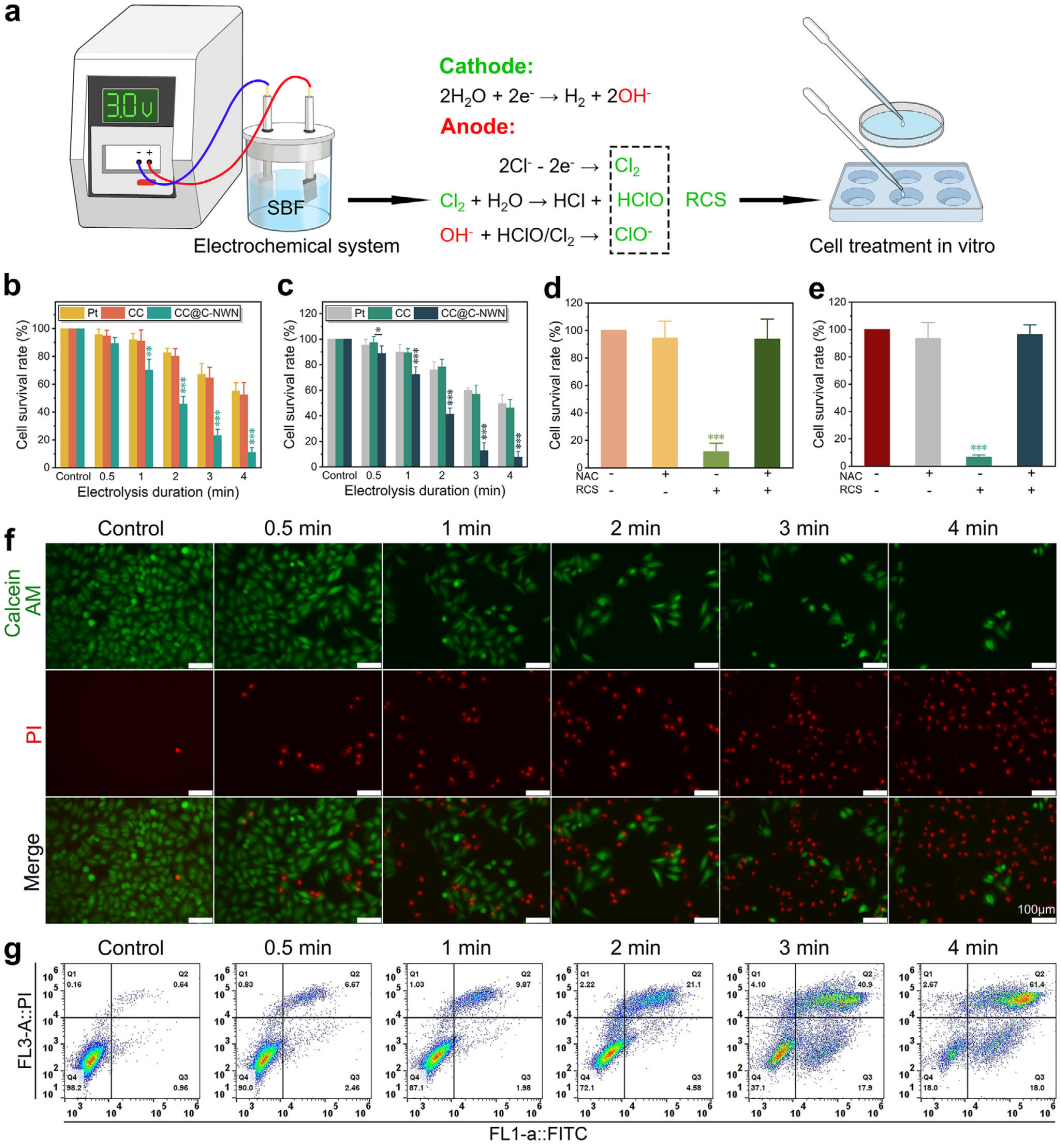
a) Illustration of the in vitro treatment of cells with the Pt, CC, and CC@C‐NWN electrochemical systems. b‐c) The CCK8 assay for treating HCCLM3 (b) and HuH7 (c) cells with the Pt, CC, and CC@C‐NWN electrochemical systems. d‐e) NAC neutralization of RCS in the CC@C‐NWN electrochemical system validated by CCK‐8 assay in HCCLM3 (d) and HuH7 (e) cells. f) Live‐dead cell staining of HCCLM3 cells treated with the CC@C‐NWN electrochemical system (Scale bar: 100 µm). g) Flow cytometric analysis of live and dead HCCLM3 cells treated with the CC@C‐NWN electrochemical system (^*^ *p* < 0.05, ^**^ *p* < 0.01, ^***^ *p* < 0.001).

Based on the efficient RCS generation capability, we further evaluated the antitumor effect of the RCS generated from the CC@C‐NWN electrochemical system on HCCLM3 cells and HuH7 cells (**Figure** **4**a). As shown in Figure 4b,c, the CCK‐8 assay revealed that the CC@C‐NWN electrochemical system exerted a dose‐dependent cytotoxic effect on HCCLM3 and HuH7 cells, a significant cytotoxicity was observed at the electrolysis duration exceeding 2 min. When the electrolysis duration reached 4 min, the 24‐h cell survival rate of HCCLM3 cells was only 11.02%, and that of HuH7 cells was only 7.61%. Moreover, the inhibitory effect of the CC@C‐NWN electrochemical system on HCCLM3 and HuH7 cells was observed to be significantly greater than that of the CC and Pt electrochemical systems, and this phenomenon became particularly evident when the electrolysis duration surpassed 2 min. Subsequently, N‐acetylcysteine (NAC) was introduced into the electrochemical system with an electrolysis duration of 4 min to neutralize the RCS.^[^ ^79^ ^]^ The results demonstrated that after the addition of NAC, the growth of tumor cells was no longer suppressed (Figure 4d,e). This phenomenon confirmed that the inhibition of tumor cells is attributed to RCS. Live‐dead staining showed that the red signal, indicating dead cells, became increasingly prominent in a duration‐dependent manner as the electrolysis duration progressed (Figure 4f). Flow cytometry analysis demonstrated that the proportion of apoptotic/dead cells increased with the prolongation of electrolysis time, which is approximately consistent with the results obtained from the CCK‐8 assay and live‐dead cell staining (Figure 4g). This significant inhibitory effect was likely attributed to the intense oxidative stress induced in tumor cells by RCS,^[^ ^80^, ^81^ ^]^ accompanied by the depletion of intracellular GSH.^[^ ^82^, ^83^ ^]^

**Figure 5:**
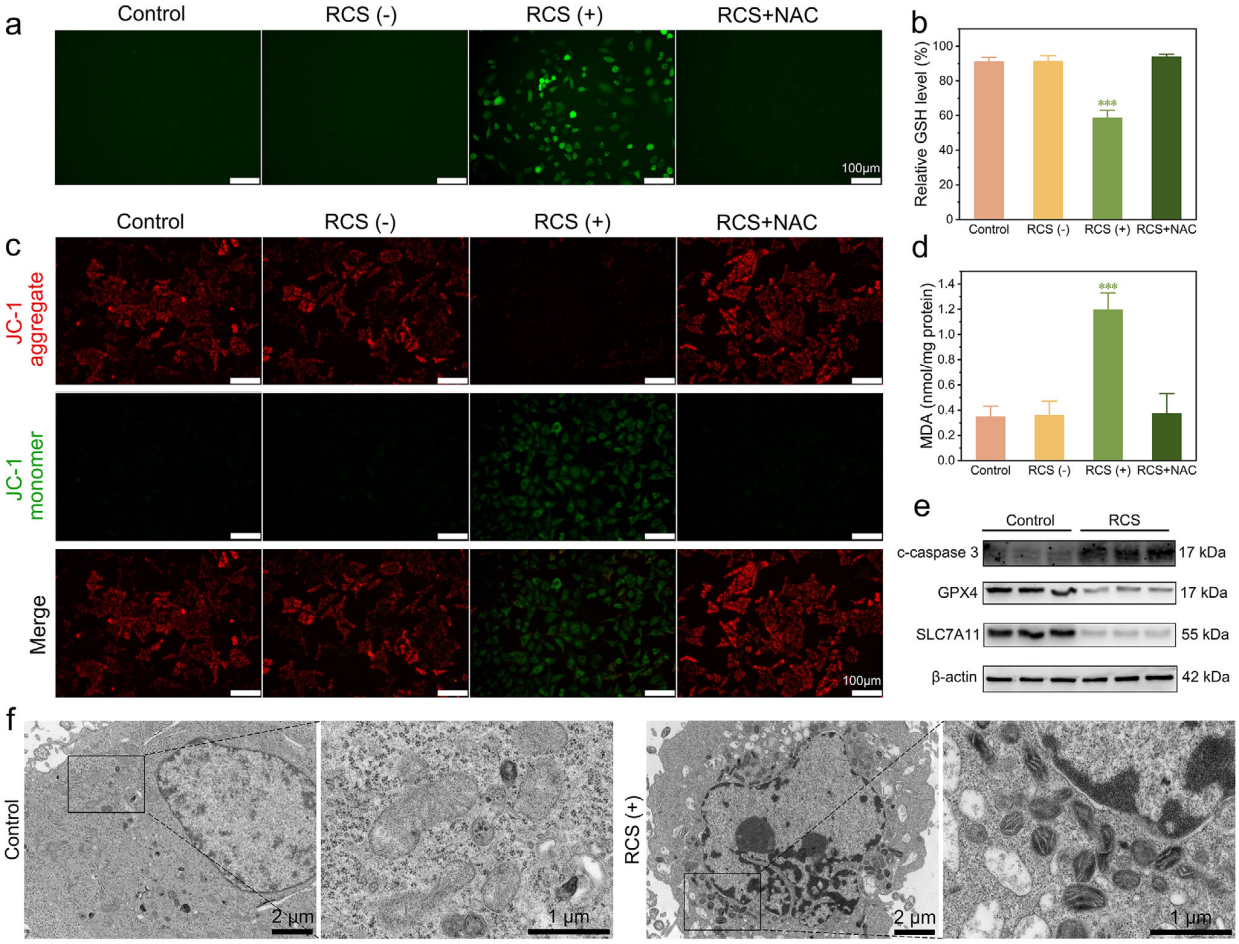
a) Fluorescence microscopy images of intracellular RCS (Scale bar: 100 µm). b) Intracellular relative GSH level. c) Fluorescence microscopy images of mitochondrial membrane potential (Scale bar: 100 µm). d) LPO products MDA level. e) Expression of c‐caspase 3, GPX4, and SLC7A11 evaluated by Western blot analysis. f) TEM image of HCCLM3 cells from the control group and RCS (+) group (^*^ *p* < 0.05, ^**^ *p* < 0.01, ^***^ *p* < 0.001).

To further validate the underlying antitumor mechanism, we conducted pertinent follow‐up experiments. Based on the results of CCK‐8 assay, we selected the CC@C‐NWN electrochemical system with an electrolysis duration of 1–2 min (RCS (+) group) for subsequent experiments on HCCLM3 cells. The detection of RCS was performed using the 2,7‐dichlorodihydrofluorescein diacetate (DCFH‐DA) probe.^[^ ^84^ ^]^ The fluorescence intensity detected by fluorescence imaging and flow cytometry indicated that the intracellular RCS levels in the RCS (+) group were significantly elevated compared to those in the control group, the CC@C‐NWN co‐incubation without electrolysis group (RCS (‐)) and the CC@C‐NWN electrochemical system neutralized by NAC group (RCS+NAC) (Figure 5a; Figures S19 and S20, Supporting Information). The strong DCF signal indicated that tumor cells are under intense oxidative stress.^[^ ^85^ ^]^ Additionally, a significant reduction in intracellular GSH levels was observed (**Figure** **5**b), indicating the disruption of redox homeostasis. To explore the oxidative damage of RCS to mitochondria, we assessed the changes in mitochondrial membrane potential through JC‐1 staining (Figure 5c; Figure S21, Supporting Information). The green fluorescence emitted by JC‐1 monomers corresponded to a low membrane potential, whereas the red fluorescence from JC‐1 aggregates indicated a high membrane potential. Analysis of the fluorescence staining patterns demonstrated that the control group, RCS (‐) group, and RCS+NAC group exhibited a predominance of red fluorescence, suggesting high mitochondrial membrane potential. In contrast, the RCS (+) group showed a predominance of green fluorescence, suggesting a reduction in mitochondrial membrane potential as a consequence of RCS treatment. We also assessed the levels of the lipid peroxidation (LPO) product malondialdehyde (MDA) and found that the MDA level in the RCS (+) group was significantly elevated compared to the control group, RCS (‐) group, and RCS+NAC group (Figure 5d). Lipids, as the primary constituents of cellular membranes, play an essential role in preserving cellular structural integrity. Excessive oxidation of lipids alters the physical characteristics of cellular membranes.^[^ ^86^ ^]^

Based on the observed mitochondrial dysfunction and GSH depletion, we hypothesized that tumor cell death might involve both mitochondria‐dependent apoptosis and ferroptosis mediated by the cyst(e)ine/ GSH/ GPX4 axis.^[^ ^87^, ^88^ ^]^ Corresponding experimental validations were subsequently conducted to investigate these mechanisms. As shown in Figure 5e and Figure S22a (Supporting Information), the elevated expression of cleaved caspase‐3 (c‐caspase 3) in the RCS (+) group indicated activation of the apoptotic pathway.^[^ ^89^ ^]^ Concurrently, the expressions of GPX4 and SLC7A11 were decreased, increasing the sensitivity of cells to ferroptosis (Figure 5e; Figure S22b, Supporting Information).^[^ ^90^ ^]^ Transmission electron microscopy (TEM) revealed hallmark ultrastructural alterations of ferroptosis in RCS (+) tumor cells, including overall mitochondrial shrinkage, reduced cristae density, simplified internal architecture, and markedly increased matrix density. These changes were consistent with the characteristics of ferroptosis (Figure 5f).^[^ ^91^ ^]^ Under the oxidative stress stimulation of RCS, the collapse of mitochondrial membrane potential triggers mitochondrial dysfunction and initiates apoptotic signaling cascades. The downstream effector molecule caspase‐3 precursor is cleaved to generate the enzyme‐active c‐caspase 3, which triggers an irreversible caspase cascade reaction and ultimately leads to cell apoptosis.^[^ ^92^ ^]^ Meanwhile, intracellular GSH depletion impairs both enzymatic activity and expression of GPX4, a critical regulator of lipid peroxidation. Oxidative stress further activates p53, which transcriptionally suppresses the cystine/glutamate antiporter SLC7A11, a key component of the Xc^−^ system, thereby diminishing cystine uptake and GSH biosynthesis.^[^ ^93^ ^]^ These coordinated mechanisms synergistically induce ferroptosis through GPX4 inactivation and RCS accumulation.

Furthermore, we evaluated the impact of the CC@C‐NWN electrochemical system on the proliferation capacity of tumor cells. The flow cytometry results indicated that the RCS treatment caused tumor cells to be arrested in the G1 phase of the cell cycle, accompanied by a decrease in Cyclin D1 protein expression, suggesting an impaired proliferative capacity of HCCLM3 cells (Figure S23, Supporting Information). Additionally, Transwell assay demonstrated that the invasive ability of RCS‐treated tumor cells are compromised (Figure S24, Supporting Information).

Interestingly, RCS treatment had a lesser impact on the viability of normal liver cells (WRL68) compared to tumor cells. This phenomenon was particularly obvious at the electrolysis time point of 2 min. However, when the electrolysis duration exceeded 3 min, the viability of WRL68 cells gradually decreased, but remained higher than that of HCCLM3 cells under the same conditions (Figure S25, Supporting Information). This phenomenon of selectively enhancing cytotoxicity in tumor cells by RCS was similar to the findings reported by Freund et al.^[^ ^94^ ^]^ This might be attributed to the fact that tumor cells are typically under more severe oxidative stress,^[^ ^2^, ^4^ ^]^ making them more susceptible to further damage by reactive species.^[^ ^5^, ^6^ ^]^

To further evaluate the biosafety of CC@C‐NWN, CC@C‐NWN were co‐cultured with HCCLM3 cells, HuH7 cells and WRL68 cells in vitro for 72 h. CCK‐8 assay demonstrated that both cell types maintained normal growth characteristics (Figure S26, Supporting Information), indicating that the CC@C‐NWN electrode exhibits no inherent cytotoxic effects.

### 2.5 Antitumor Effect of CC@C‐NWN in vivo

**Figure 6:**
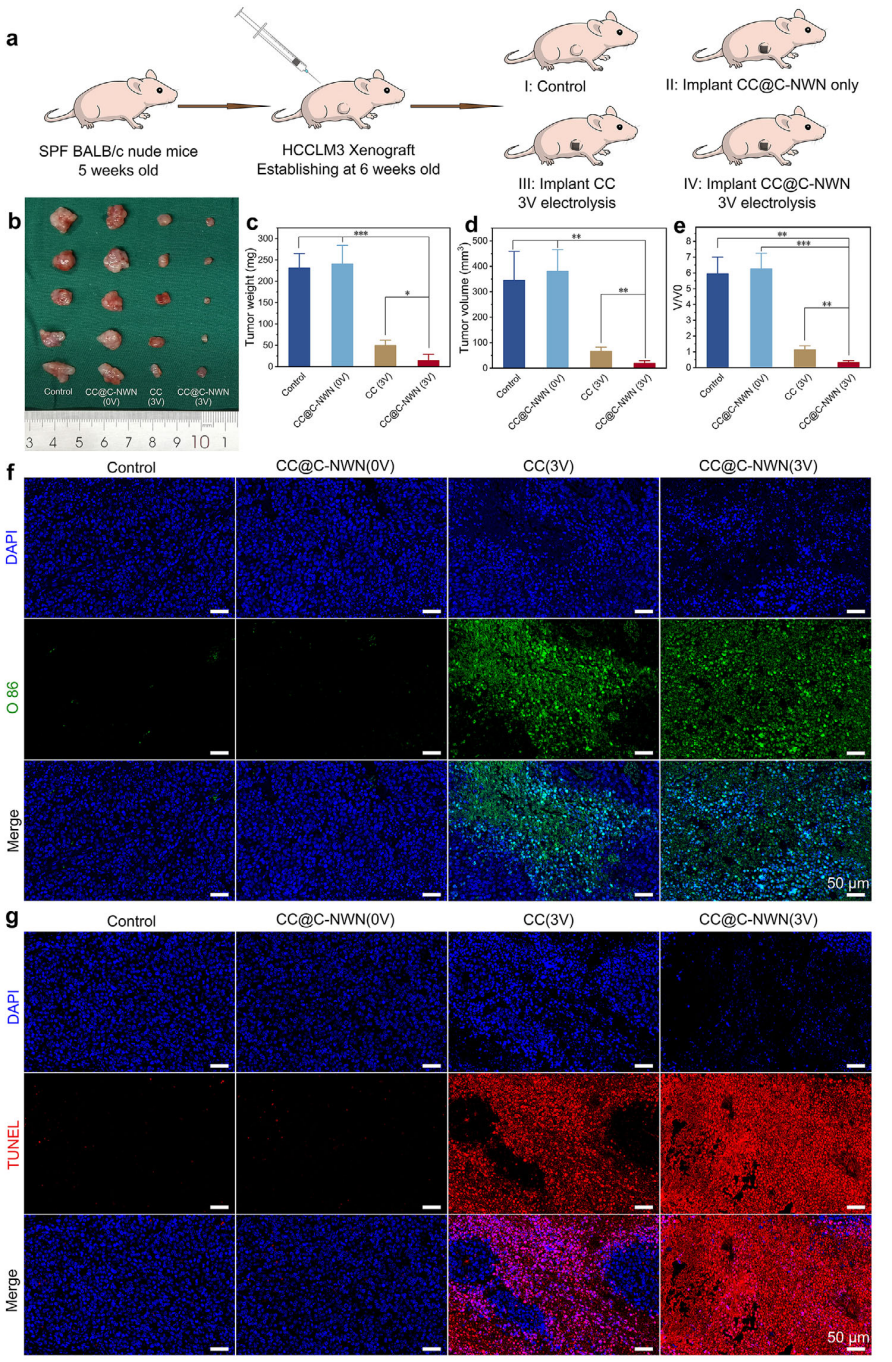
a) The treatment procedure in vivo. b) Tumor specimens retrieved from each group. c) Tumor weight for each group. d) The volume of tumor specimens in each group. e) Relative tumor volumes for each group. f) O86 staining of tumor sections. g) TUNEL staining of tumor sections (Scale bar: 50 µm) (^*^ *p* < 0.05, ^**^ *p* < 0.01, ^***^ *p* < 0.001).

Based on the superior antitumor effect of RCS generated by CC@C‐NWN in vitro, we subsequently explored the antitumor effect of CC@C‐NWN in the HCCLM3 xenograft tumor BALB/c nude mice model. When the subcutaneous tumors reached ≈60 mm^3^, the model mice were randomly divided into 4 groups: I: control group, II: CC@C‐NWN (0 V) group, III: CC (3 V) group, and IV: CC@C‐NWN (3 V) group (**Figure** **6**a). CC and CC@C‐NWN were trimmed into a dumbbell shape, one end was implanted subcutaneously and clung to the tumor, the other end was exposed externally for attaching to the DC power supply electrode clip (Figure S27, Supporting Information). Mice in the control group and the CC@C‐NWN (0 V) group exhibited significantly rapid tumor growth (Figure 6b–e), and the relative tumor volume (V/V0) were 5.94 and 6.25, respectively. The tumor growth in the CC (3 V) group was suppressed, and the volume remained basically stable at pre‐treatment level (V/V0 = 1.13). The CC@C‐NWN (3 V) treatment demonstrated a significant anti‐tumor effect, and the relative tumor volume (V/V0 = 0.31) exhibited a marked reduction. This significant anti‐tumor effect of CC@C‐NWN could be attributed to the superior RCS‐catalyzing performance.

We used the HClO/ClO^−^ specific fluorescent probe O86 to detect the distribution of RCS in the tumor tissues of each group of mice during the treatment process. As shown in Figure 6f, no significant green RCS fluorescent signals (O86 signal) were observed in tumor tissues of the control group and CC@C‐NWN (0 V) group, while densely distributed DAPI signals were detected. In the CC (3 V) group, enhanced O86 signals began to emerge but exhibited heterogeneous distribution. In contrast, tumor tissues in the CC@C‐NWN (3 V) group demonstrated intensified O86 signals that were uniformly distributed throughout the sample regions. This indicated that the tumor tissue in the CC@C‐NWN group achieved the most complete RCS distribution during the treatment process. In the TUNEL staining, the tumor tissues of the control group and the CC (0 V) group showed uniform DAPI signals, with no obvious TUNEL signals. In the CC (3 V) group, enhanced TUNEL signals began to appear in some tissue areas, while the DAPI signals started to weaken and disappear. In the CC@C‐NWN (3 V) group, the tumor tissues exhibited the strongest TUNEL signals and the weakest DAPI signals (Figure 6g). The TUNEL fluorescence staining can be mutually corroborated with the O86 staining results, which fully confirm that the CC@C‐NWN electrode achieved optimal therapeutic efficacy under 3 V DC drive. Histopathological analysis of the hematoxylin and eosin (H&E) – stained tissue sections from the mice tumor revealed no obvious areas of cell death in the control and the CC@C‐NWN (0 V) groups. However, in the CC (3 V) group and the CC@C‐NWN (3 V) group, partial regions of the sections exhibited marked structural disruption. In these areas, cells exhibited signs of shrinkage. The nuclear staining became more deeper, indicating chromatin condensation. The cytoplasm appeared condensed and exhibited eosinophilic changes. These cytological alterations were more pronounced in the CC@C‐NWN (3 V) group (Figure S28, Supporting Information). Furthermore, immunohistochemical staining analysis revealed that the tumor tissues treated with CC@C‐NWN (3 V) exhibit the weakest Ki67 staining signal (Figure S29, Supporting Information). These findings indicated that CC@C‐NWN (3 V) can maximally promote cell death and inhibit cell proliferation.

### 2.6 Biosafety Validation of CC@C‐NWN in vivo

At the end of the treatment process, the skin condition at the treatment site of the mice was normal, no signs of redness, swelling, or discharge at the incision sites were observed (Figure S30, Supporting Information). Subsequently, the electrodes and residual tumor tissues were carefully removed. The skin was sutured using 4‐0 sutures, and the wound area was regularly disinfected with 75% medical alcohol. On the 8th postoperative day, the sutures were removed. Three days after suture removal, the skin was examined, revealing excellent wound healing without any apparent complications (Figure S31, Supporting Information). Mice in the CC@C‐NWN (0 V), CC (3 V), and CC@C‐NWN (3 V) groups all experienced an acute but mild weight loss on the first day following material implantation surgery (Figure S32a, Supporting Information), this phenomenon might be attributed to the short‐term stress from the surgical procedure and anesthesia. Throughout the subsequent experimental period, the body weight of the mice gradually recovered. Additionally, blood samples of the mice were collected for biochemical analysis. As expected, the alanine aminotransferase (ALT), aspartate aminotransferase (AST), creatinine (CREA), and blood urea nitrogen (BUN) levels did not exhibit significant difference among the different groups (Figure S32b–e, Supporting Information). To further evaluate the biocompatibility of CC@C‐NWN, histological examinations of the major organs from mice in each group were performed using the H&E staining. The results demonstrated that the histological structure and morphology of the heart, liver, spleen, lung, and kidney tissues exhibited no obvious abnormality (Figure S33, Supporting Information). These results further confirmed the excellent biocompatibility of CC@C‐NWN. On one hand, this specified the advantage of the inherent nontoxicity of carbon‐based materials. On the other hand, short‐term local treatment also indirectly supported the perspective that HOCl is an endogenous substance that can be tolerated by all mammals at low concentrations.^[^ ^95^ ^]^

## 3 Conclusion

In general, the flexible carbon‐based anodic catalytic electrode (CC@C‐NWN) that we designed demonstrated excellent effect in inhibiting tumor growth. This significant tumor‐suppressing efficacy was attributed to the efficient RCS production capability of CC@C‐NWN. The CC@C‐NWN exhibited superior hydrophilicity, thus enhanced the ion conduction efficiency and increased the utilization rate of the electrode. The high specific surface area and electrochemical surface area of the electrode offered more reactive sites for the reaction with Cl⁻. Additionally, the nanostructure of CC@C‐NWN enhanced the Cl⁻ concentration gradient at the electrode‐electrolyte interface. Moreover, the presence of carbon vacancies within the C‐NWN boosted the Cl⁻ adsorption, and the theoretical overpotential for CER was reduced more significantly than that for OER, thus facilitating the CER process. The CC@C‐NWN electrochemical system led to a substantial increase in RCS level within the tumor cell, disrupting redox homeostasis and reducing the mitochondrial membrane potential. Consequently, both ferroptotic and apoptotic signaling pathways were activated, resulting in a synergistic anti‐tumor effect through apoptosis and ferroptosis. in vivo, the CC@C‐NWN catalytic electrode demonstrated the ability to significantly inhibit tumor growth under low‐voltage (3 V) condition. Therefore, the CC@C‐NWN electrode provided a promising strategy for RCS‐based tumor treatment.

## 4 Experimental Section

### Preparation of CC@C‐NWN

The synthesis of CC@C‐NWN was achieved by in situ electrodeposition of PPy nanowire network on the surface of bare CC fibers (CC@PPy‐NWN), followed by high‐temperature carbonization under a nitrogen atmosphere. Specifically, commercial CC (Ce Tech Co., Ltd, Taiwan, China) was cut according to 1.5 × 1.5 cm and cleaned with acetone, ethanol, and ultrasonic cleaning in deionized water for 30 min to remove oil and impurities, and then dried in a 60 °C oven. To avoid the influence of materials quality on experimental results, all commercial CC used in this work were from the same batch and subjected to identical pretreatment methods. Next, the dried CC was placed in the porcelain combustion boat and heat treated for 2 h at 550 °C in the air atmosphere in the tube furnace to improve the hydrophilicity of the CC. An electrolyte containing phosphate buffer solution, sodium p‐toluenesulfonate dopant, and pyrrole monomer was configured. The CC@PPy‐NWN composite was formed by electrodeposition at a constant voltage of 0.85 V for 30 min, using pre‐treated CC as the working electrode, a Pt electrode as the counter electrode, and an Ag/AgCl electrode as the reference electrode. The CC@PPy‐NWN was washed with deionized water, dried, and then placed in the tube furnace for thermal treatment at 800 °C under a nitrogen atmosphere for 2 h to obtain CC@C‐NWN.

### EPR Testing

Instrument model: Bruker EMXplus‐6/1. According to the material sequence numbers, the samples to be tested were directly weighed and placed into quartz tubes. Then, the quartz tubes were put into the resonant cavity of the instrument, ensuring that the samples (at the bottom of the quartz tubes) were located at the center of the resonant cavity. After that, the tuning and scanning tests were initiated.

### XRD Testing

The powder sample was placed on a glass slide and tested using an X‐ray diffractometer. The model of the instrument was Rigaku SmartLab SE from Japan. The testing range was 5–90° (as per the test requirements), with a scanning speed of 2° per min (as per the test requirements). The radiation source was Cu‐Kα rays, the tube voltage was 40 kV, and the current was 40 mA.

### XPS Testing

Took an appropriate amount of sample and pressed it into a sheet, then attached it to the sample tray. Placed the sample in the sample chamber of the Thermo Scientific K‐Alpha XPS instrument. When the pressure in the sample chamber is less than 2.0 × 10^−7^ mbar, transfer the sample to the analysis chamber. The spot size is 400 µm, the working voltage is 12 kV, and the filament current is 6 mA. The full‐spectrum scan pass energy is 150 eV with a step size of 1 eV; the narrow‐spectrum scan pass energy is 50 eV with a step size of 0.1 eV.

### BET Testing

The sample was pretreated for 8 h under vacuum at 120 °C using the standard degassing station of the Microlab instrument. Then, the nitrogen adsorption‐desorption test was conducted on the sample at 77 K liquid nitrogen conditions using the 4‐station fully automatic specific surface area analyzer of the APSP 2460 model from Micromeritics, USA. After the instrument analysis was completed, the isothermal adsorption‐desorption curve was obtained, and the total specific surface area of the material was calculated by the BET method.

### SEM Testing

Took a small amount of sample and directly stuck it onto the conductive adhesive. Then, used the Quorum SC7620 sputtering coater to spray gold for 45 s (the specific gold spraying time is determined according to the sample / test requirements), with a current of 10 mA. Subsequently, used the ZEISS Sigma 300 scanning electron microscope to take pictures of the sample morphology and perform energy spectrum mapping tests, etc. The acceleration voltage was 3 kV when taking pictures of the morphology, and 15 kV when performing energy spectrum mapping. The detector used is the SE2 secondary electron detector.

### Electrochemical Testing

The measurements of the electrocatalytic CER were conducted in a three‐electrode glass cell at room temperature using the Bio‐Logic VSP electrochemical workstation. An Ag/AgCl (3.5 M KCl solution) (Shanghai Chuxi Instruments) electrode served as the reference electrode. A platinum plate electrode (2×2 cm) was used as the counter electrode. Pt (1.5 × 1.5 cm), CC (1.5 × 1.5 cm), or CC@C‐NWN (1.5 × 1.5 cm) was used as the working electrode. SBF (Yuanye Bio‐Technology Co., Ltd., Shanghai, China) served as the electrolyte, supplied chloride ions for the electrolysis process. LSV curves were measured at a scan rate of 5 mV s^−1^ over a potential range of 0 V to 3.5 V versus Ag/AgCl. Potentials were calibrated using a reversible hydrogen electrode (RHE), utilizing the Nernst equation: E(RHE) = E(Ag/AgCl) + 0.0952× pH + 0.2046. When calculating the Tafel slope, the overpotential was converted as follows: η(CER) = E(RHE) – 1.36. CV scans for the electrochemical C_dl_ were conducted within a potential window of 1.1 to 1.7 V versus Ag/AgCl, with a series of scan rates ranging from 20 to 120 mV s^−1^ in increments of 20 mV s^−1^. The stability test was conducted at a current density of 10 mA cm^−2^ for 50 h.

### DFT Calculation

All first‐principles DFT calculations were performed using the Vienna Ab initio Simulation Package (VASP) based on the projector‐augmented wave (PAW) method.^[^ ^96^ ^]^ The electron‐ion interaction and the exchange‐correlation energy were treated at the generalized gradient approximation (GGA) level with the Perdew—Burke–Ernzerhof (PBE) functional.^[^ ^97^ ^]^ The long‐range effects of nonlocal van der Waals interactions were adopted with the DFT‐D3 dispersion corrections method.^[^ ^98^ ^]^ As shown in Figure S34 (Supporting Information), the convergence tests were performed on the cutoff energy and k‐point mesh. According to the results, the plane wave basis set was affixed with a kinetic energy cut‐off set at 520 eV, and the geometries were sampled with a (3 × 3 × 1) k‐point mesh. A vacuum layer of 15 Å on the z‐axis was set to avoid interaction between carbon layers. All atoms were allowed to relax till the atomic forces were smaller than 1×10^−3^ eV Å^−1^, and the electronic self‐consistent convergence criterion was lower than 10^−3^ eV atom^−1^, respectively. Electronic property calculations were carried out to understand the charge transfer mechanism in CER including Bader charge calculation of charges on C atoms and Cl atoms. The ELF plots were made using the VESTA software.^[^ ^99^ ^]^

The electrocatalytic CER leads to the formation of *Cl intermediate. The adsorption energies (ΔE) of the intermediate and its corresponding Gibbs free energies of adsorption (ΔG) are calculated using the following equations:

(1) $$\Delta E\;\left(*\mathrm{Cl}\right)=E\;\left(*\mathrm{Cl}\right)-E\;\left(*\right)-0.5E\;\left(Cl_{2}\right)$$

(2) ΔG=ΔE+ΔZPE−TΔS

where ΔZPE represents the change in zero‐point vibrational energy, and TΔS represents the entropy contribution at room temperature. According to the computational electrode model proposed by Nørskov et al.,^[^ ^100^ ^]^ the Gibbs free energy as a function of pH and standard potential (U) of the reversible chlorine electrode referenced to a standard hydrogen electrode (SHE), i.e., 1.36 V at 298 K, is defined as:

(3) $$\Delta G*\mathrm{Cl}\;\left(\mathrm{pH},\;U\right)=\Delta E*\mathrm{Cl}+\Delta \mathrm{ZPE}-T\Delta S-\mathrm{eU}+1.36$$

The OER usually follows the scheme developed by Nørskov et al. The OER is assumed to contain four elementary reaction steps and each step involves electron transfer accompanied by proton expulsion, as follow:^[^ ^101^, ^102^ ^]^

(4) $$H_{2}O\;\left(\mathrm{aq}\right)+*\to \mathrm{HO}*+H^{+}+e^{-}$$

(5) $$\mathrm{HO}*\to O*+H^{+}+e^{-}$$

(6) $$O*+H_{2}O\;\left(\mathrm{aq}\right)\to \mathrm{HOO}*+H^{+}+e^{-}$$

(7) $$\mathrm{HOO}*\to *+O_{2}\left(g\right)+H^{+}+e^{-}$$

where the symbol * denotes the model. Accordingly, we can get the reaction free energy formula for the intermediate states (U (versus RHE) = 0):

(8) $$\Delta G_{1}=G\;\left(\mathrm{HO}*\right)+G\;\left(H^{+}\right)+\mu \;\left(e^{-}\right)-G\;\left(*\right)-G\;\left(H_{2}O\right)$$

(9) $$\Delta G_{2}=G\;\left(O*\right)+2G\;\left(H^{+}\right)+2\mu \;\left(e^{-}\right)\;G\;\left(*\right)-G\;\left(H_{2}O\right)$$

(10) $$\Delta G_{3}=G\;\left(\mathrm{HOO}*\right)+3G\;\left(H^{+}\right)+3\mu \;\left(e^{-}\right)-G\;\left(*\right)-2G\;\left(H_{2}O\right)$$

(11) $$\Delta G_{4}=\mathrm{Dg}\;\left(*+O_{2}\right)=4.92-\left(dG_{1}+dG_{2}+dG_{3}\right)$$

The *η*_OER_ can be defined as follows:

(12) $$\eta_{\mathrm{OER}}=\max\;\left(\Delta G_{1},\;\Delta G_{2},\;\Delta G_{3},\;\Delta G_{4}\right)/e-1.23\;V$$

### COMSOL Simulation—Transport Model

The ion transport in the solution of the Cl⁻ ion consumption unit is described by the Nernst–Planck–Poisson equation. The ion flux density *j_i_* of the Cl⁻ solution is:

(13) $$j_{i}=D_{i}\left(\nabla c_{i}+\frac{z_{i}Fc_{i}}{RT}\nabla \varphi \right)$$

In the equation, *j_i_* is the ion mass flow diffusion vector (mol m⁻^2^ s⁻^1^), *D_i_* is the ion diffusion coefficient (m^2^ s⁻^1^), *c_i_* is the ion concentration (mol m⁻^3^), *z_i_* is the ion valence, *F* is the Faraday constant, *R* is the ideal gas constant, *T* is the temperature in the unit (K), and *φ* is the electric potential (V).

The dilute mass–transfer field was used to describe the transfer and concentration distribution of various component ions in the unit due to convection and diffusion; the electrostatic field was used to solve the distribution of the electric field and electric potential in the unit. The two fields were coupled by means of potential coupling and space–charge–density coupling, that is, the Nernst – Planck equation describing the mass transfer of all ions and the Poisson equation describing the charge density and the electric field are coupled.

### COMSOL Simulation—Boundary Conditions

The boundary condition for the boundary concentration of the Cl⁻ solution unit is:

(14) $$c^{j}=c_{0,j}$$

(15) $$c_{0,j}=147.8\;\left(\mathrm{mol}/m^{3}\right)$$

The normal component of the ion flux at the boundary is zero:

(16) $$\vec{n}\cdot j=0$$

### Cell Culture

HCCLM3 (Human High Metastatic Potential Hepatocellular Carcinoma Cell Line) cells were purchased from the Shanghai Cell Resource Center, Chinese Academy of Sciences (SCRC, CAS), the WRL68 (Human Hepatic Cell Line) cells and the HuH7 (Human Hepatoma Cell Line) cells were purchased from iCell Bioscience Inc (Shanghai, China). Roswell Park Memorial Institute (RPMI‐1640), Dulbecco's Modified Eagle's Medium (DMEM, high glucose) and 0.25% trypsin‐EDTA was purchased from Gibco (USA), fetal bovine serum (FBS) was supplied by Shuangru Biotechnology Co., Ltd. (Suzhou, China). Penicillin‐streptomycin solution was purchased from Servicebio Technology Co., Ltd. (Wuhan, China). The culture medium used for cell culturing consisted of DMEM/RPMI‐1640 supplemented with 10% FBS and 1% penicillin‐streptomycin solution. Cells were incubated in a humidified incubator with an atmosphere containing 5% CO_2_ at a temperature of 37 °C.

### Cell Viability

Cell viability was assessed using the Cell counting Kit‐8 (CCK8) and the Calcein ‐AM/PI Live/Dead Cell Double Stain Kit (Servicebio Technology Co., Ltd., Wuhan, China), following the manufacturer's instructions. The CCK‐8 working solution was prepared by mixing CCK‐8 solution with culture medium in a volume ratio of 1:10. CCK‐8 working solution were added to the cells and incubated in a cell culture incubator for 1–4 h. The absorbance at 450 nm was then measured using a microplate reader. The cell survival rate was calculated using the following equation:

(17) $$\begin{array}{ccc} & & \left(\mathrm{OD}\;\mathrm{valu}e_{\left(\mathrm{experimental}\right)}-\mathrm{OD}\;\mathrm{valu}e_{\left(\mathrm{blank}\right)}\right)\\ & & /\left(\mathrm{OD}\;\mathrm{valu}e_{\left(\mathrm{control}\right)}-\mathrm{OD}\;\mathrm{valu}e_{\left(\mathrm{blank}\right)}\right) \end{array}$$

For Live/Dead cell staining. Cells were washed with phosphate‐buffered saline (PBS) 2–3 times. Subsequently, 0.2 µL of Calcein‐AM reagent and 0.2 µL of PI reagent were added to each well, followed by 100 µL of assay buffer. The samples were incubated in the dark at 37 °C in a cell culture incubator for 15–30 min, and then observe under a fluorescence microscope.

### Flow Cytometry Assay

Apoptosis in HCCLM3 cells was evaluated using the Annexin V‐FITC/PI Apoptosis Detection Kit (Servicebio Technology Co., Ltd., Wuhan, China). After the corresponding treatment, cells were harvested, washed twice with PBS, and stained with Annexin V‐FITC/PI for 15 min. Subsequently, the stained cells were transferred to flow cytometry tubes using a filter net and analyzed for apoptotic rate using a flow cytometer.

The detection of RCS was performed using the DCFH‐DA fluorescence probe (Beyotime Biotechnology Co., Ltd., Shanghai, China). The DCFH‐DA was diluted to a concentration of 10 µM with serum‐free culture medium and added to the cells for incubation at 37 °C in a cell culture incubator for 20 min to load the DCFH‐DA probe. After incubation, the cells were washed three times with serum‐free cell culture medium. Following probe loading, the cells were subjected to the corresponding treatments. The cells were then collected and analyzed using a flow cytometer to detect the fluorescence intensity.

Cell cycle analysis was performed using the PI/RNase reagent (Becton, Dickinson and Company, USA). After treatment, the cells were digested, centrifuged, and fixed overnight with pre‐chilled 70% ethanol. The cells were then washed twice with PBS. Following staining with the PI/RNase reagent, the cells were analyzed using a flow cytometer.

### Western Blot Analysis

The HCCLM3 cells were lysed using radioimmunoprecipitation assay (RIPA) lysis buffer at 4 °C for 30 min, then the total proteins were extracted for Western blot analysis. The samples were denatured at 100 °C for 10 min. Electrophoresis was conducted using a 12% sodium dodecyl sulfate‐polyacrylamide gel (SDS‐PAGE), followed by transfer to a polyvinylidene fluoride (PVDF) membrane. The membrane was then blocked with 5% nonfat milk for 1 h, and incubated with the corresponding primary and horseradish peroxidase (HRP)‐conjugated secondary antibodies. Finally, the signals of the protein bands were detected by chemiluminescence.

### RCS Fluorescence Staining

The procedure followed the steps of flow cytometry analysis, with the exception of cell digestion. After completing the corresponding steps, the cells were observed under a fluorescence microscope.

### Intracellular Relative GSH Level

GSH levels were measured using a GSH and GSSG Assay Kit (Beyotime Biotechnology Co., Ltd., Shanghai, China) according to the manufacturer's instruction. After subjecting the cells to the corresponding treatments, they were collected and treated with three volumes of protein removal reagent. Subsequently, the samples underwent rapid freeze‐thaw cycles using liquid nitrogen and a 37 °C water bath, followed by centrifugation to obtain the supernatant for analysis. The absorbance was measured at 405 nm using a microplate reader. GSH content was determined using the formula: GSH = Total Glutathione – 2 × oxidized glutathione disulfide (GSSG). The relative GSH content was then expressed as the ratio of GSH to total glutathione content.

### Malondialdehyde (MDA) Level Measurement

The MDA level was measured using a Lipid Peroxidation MDA Assay Kit (Beyotime Biotechnology Co., Ltd., Shanghai, China). According to the manufacturer's instruction, cells that had undergone the corresponding treatments were lysed with Cell lysis buffer for Western and IP (Beyotime Biotechnology Co., Ltd., Shanghai, China), and the supernatant was collected after centrifugation for further analysis. The prepared MDA detection working solution was added to the samples, which were then heated at 100 °C for 15 min, cooled to room temperature, and the absorbance was measured at 532 nm using a microplate reader. Protein content in the samples was determined using a protein quantification kit. The MDA content in the samples was expressed as the amount of MDA per unit weight of protein.

### Mitochondrial Membrane Potential Detection

Mitochondrial membrane potential was assessed using the JC‐1 Mitochondrial Membrane Potential Assay Kit (Servicebio Technology Co., Ltd., Wuhan, China). Following the manufacturer's instructions, post‐treatment cells were washed twice with JC‐1 buffer and then incubated with the prepared JC‐1 working solution in a CO_2_ incubator protected from light for 15 min. After incubation, the cells were washed again with JC‐1 buffer and observed under a fluorescence microscope.

### Cell Invasion Assay

To evaluate the invasive capabilities of treated cells, we performed a Transwell assay. Matrigel (Servicebio Technology Co., Ltd., Wuhan, China) was mixed with pre‐chilled serum‐free medium at a ratio of 1:8 and added to the upper chamber of the Transwell insert. The cells were starved in serum‐free medium for 24 h. A 200 µL suspension of cells was added to the upper chamber, while 500 µL of complete medium was added to the lower chamber, and the setup was incubated in a CO_2_ incubator for 48 h. Subsequently, the cells were fixed with 4% paraformaldehyde and stained with 0.1% crystal violet. Invasion were observed and quantified under an inverted microscope.

### Construction and Treatment of Xenograft Tumors

Five‐week‐old male BALB/c nude mice were purchased from Beijing Vital River Laboratory Animal Technology Co., Ltd. The experimental design, procedures, and methods of euthanasia for the animal experiments were reviewed and approved by the Laboratory Animal Welfare and Ethics Committee of the Army Medical University (AMUWEC20245277), complying with ethical standards and animal welfare requirements. The mice were housed in an SPF‐grade facility for one week to acclimate. Subsequently, each mouse was subcutaneously injected with 5 × 10^6^ HCCLM3 cells under the armpit. The formula for calculating tumor volume was: (length × width^2^)/2. Once the tumors reached a volume of ≈60 mm^3^, the mice were grouped and subjected to surgical procedures for the implantation of materials. The day of implantation surgery was designated as Day 0, with no electrotherapy treatment administered.

CC or CC@C‐NWN was cut into a dumbbell shape with enlarged ends. One end, measuring ≈5 × 5 mm (Figure S27, Supporting Information), was designed for subcutaneous implantation. The surgical procedure for implanting the material was as follows: Under a laminar flow hood, sterile towels were laid, and mice were anesthetized with 1% pentobarbital sodium. The tumor area and surrounding skin were disinfected, and sterile drapes were applied to expose the tumor site. A tissue scissor was used to make a 5 mm incision in the skin above the tumor. One end of the prepared CC/CC@C‐NWN was inserted into the subcutaneous tissue of the tumor area and adhered to the tumor, and the skin incision was closed with two interrupted sutures using 4‐0 monofilament sutures. At the end of the procedure, the incision area was disinfected again, and the incision was covered with gauze and secured with adhesive tape during non‐treatment periods. The wound dressing was kept clean and dry, with regular disinfection. In case of any abnormal conditions such as redness, swelling, or discharge at the incision site, sutures were removed and managed promptly.

The control group received no treatment, while the CC@C‐NWN (0 V) group was implanted with the material without electrotherapy. The CC (3 V) group and CC@C‐NWN (3 V) group were treated with 3 V DC, using a Pt needle (diameter 0.5 mm, Boyan Technology Co., Ltd., Taizhou, Jiangsu) as the cathode, which was inserted into the subcutaneous tissue of the tumor area, close to the anode. As shown in Figure S27b (Supporting Information), the electrotherapy lasted for 2 min per session, once a day for 5 days, with a 3‐day observation period. Under anesthesia, blood was collected from the orbital sinus, and the mice were euthanized with an overdose of anesthesia. The heart, lungs, liver, spleen, kidneys, and tumor tissues were harvested for subsequent examinations.

### H&E Staining

The paraffin sections were dewaxed, stained with hematoxylin solution for 5–10 min, and then stained with eosin solution for 30–60 s. Subsequently, they underwent gradient ethanol dehydration treatment, were placed in xylene for 30 s for transparency processing, and finally sealed with neutral resin.

### TUNEL Staining

The sections were dewaxed, followed by proteinase K digestion. Then, the membrane was disrupted by adding the membrane disruption working solution. TDT enzyme, dUTP and buffer were added, and the nuclei were counterstained with DAPI staining solution. Finally, the sections were mounted with anti‐fluorescence quenching mounting medium. DAPI emits at 420 nm under ultraviolet excitation range (330‐380 nm), showing blue fluorescence; TMR emits at 590 nm when excited at 510–561 nm, showing red fluorescence.

### Ki67 Immumohistochemical Staining

The sections were dewaxed and hydrated. Antigen retrieval was performed by heating in EDTA 8.0 for 30 min. The sections were then immersed in a 3% hydrogen peroxide solution to block endogenous peroxidase, followed by the addition of 3% BSA. Primary and secondary antibodies were successively added. Subsequently, DAB staining was carried out. The cell nuclei were counterstained with hematoxylin for ≈3 min, and then blued with hematoxylin bluing solution. Finally, the tissues were dehydrated and mounted. Blue represents the hematoxylin‐stained cell nuclei, and brownish‐yellow indicates the positive expression shown by DAB.

### O86 Staining

De‐paraffinize the sections, add 200 µL of the washing working solution, and let it stand for 10 min. Add 100 µL of the staining working solution, and incubate in the 37 °C incubator away from light for 30 min. Wash the sections twice with PBS, observe under an upright fluorescence microscope and collect images.

### Statistical Analysis

All results presented in this article were expressed as the mean ± standard deviation (mean ± SD). Graphs were generated using OriginPro 2022, and statistical analyses were performed using SPSS 26.0. Significant differences between the two samples were analyzed using the independent samples Student's t‐test. Significant differences among multiple groups were analyzed using one‐way analysis of variance (ANOVA). P‐value <0.05 was considered to indicate statistical significance (^*^ *p*<0.05, ^**^ *p*<0.01, ^***^ *p*<0.001).

### Ethical Statement

The experimental design, procedures, and methods of euthanasia for the animal experiments were reviewed and approved by the Laboratory Animal Welfare and Ethics Committee of the Army Medical University (AMUWEC20245277), complying with ethical standards and animal welfare requirements.

## Conflict of Interest

The authors declare no conflict of interest.

## Supporting information



Supporting Information

## Data Availability

The data that support the findings of this study are available from the corresponding author upon reasonable request.
