# Supplementary material for: Carbon‐Based Flexible Electrode for Efficient Electrochemical Generation of Reactive Chlorine Species in Tumor Therapy
Source: Adv Healthc Mater. 2025 May 24;14(17):2500369. doi: 10.1002/adhm.202500369 (PMC12232165; doi:10.1002/adhm.202500369)
Supplement: Supplementary file 1 — Supporting Information [file ADHM-14-0-s001.docx]

Supporting Information

Carbon-Based Flexible Electrode for Efficient Electrochemical Generation of Reactive Chlorine Species in Tumor Therapy

*Cuinan Jiang ^a,1^, Zhaoyu Chen ^b,1^, Ruihao Yang ^a,1^, Ziga Luogu ^b^, Qian ren ^a^, Hao Hu ^a^, Kaixin Wang ^b^, Senlin Li ^a^, Changlin Deng ^a^, Meng Li ^b,^*, Lu Zheng ^a,^**

**Supplementary tables and figures**

**Table S1.** Ionic composition of human plasma and simulated body fluid (SBF) (mmol/L).

| ion | Na^+^ | K^+^ | Cl^-^ | Mg^2+^ | Ca^2+^ | $\mathrm{HPO}_{4}^{2-}$ | $\mathrm{SO}_{4}^{2-}$ |
| --- | --- | --- | --- | --- | --- | --- | --- |
| Human plasma | 142.0 | 5.0 | 118.0 | 1.5 | 2.5 | 1.0 | 0.5 |
| SBF | 142.0 | 5.0 | 147.8 | 1.5 | 2.5 | 1.0 | 0.5 |

The numerical values of the parameters required for the calculation of the Cl⁻ ion consumption unit model are shown in **Table S2-S4**:

**Table S2.** Model Parameters.

| Structural Parameter | Width of Solution Unit (μm) | 50 |
| --- | --- | --- |
|  | Height of Solution Unit (μm) | 50 |
|  | Fiber Diameter (μm) | 30 |
|  | Nanowire Length (μm) | 2 |

**Table S3.** Physical Parameter.

| Physical Parameter | Initial Concentration of Cl⁻ Ions (mol/m³) | 147.8 |
| --- | --- | --- |
|  | Fiber Potential (V) | 1.36 |

**Table S4.** Material Parameter.

| Material Parameter | Charge of Cl⁻ | -1 |
| --- | --- | --- |
|  | Diffusion Coefficient of Cl⁻ (m²/s) | 1×10^-9^ |

**Table S5.** The adsorption energy of *Cl and *OCl on C and C_v_ model.

|  | E(*Cl) (eV) | E(*OCl) (eV) |
| --- | --- | --- |
| C | 0.402 | 1.80 |
| Cv | -2.31 | 1.20 |


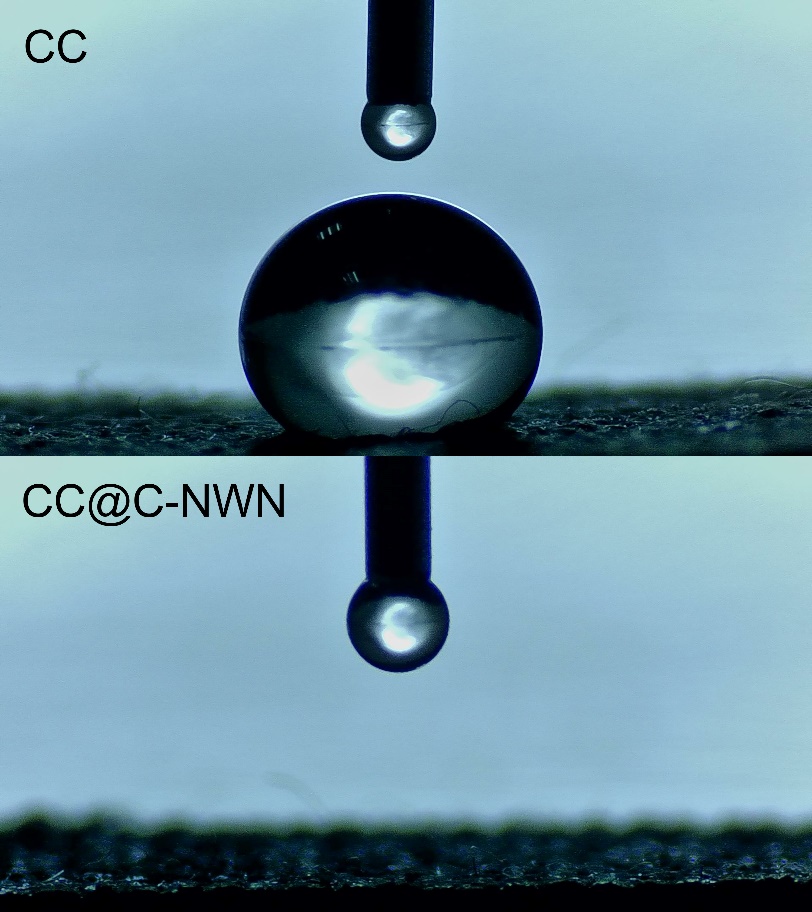


**Figure S1.** The hydrophilicity of CC and CC@C-NWN. CC exhibited a contact angle of approximately 132°. CC@C-NWN demonstrated impressive hydrophilicity with a contact angle of 0°.


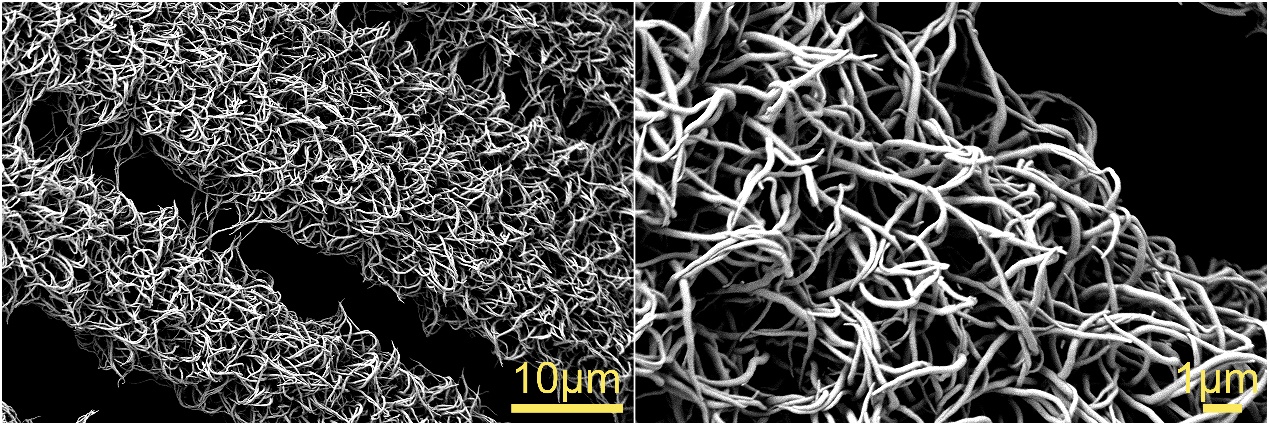


**Figure S2.** SEM images of the PPy-NWN nanostructure.


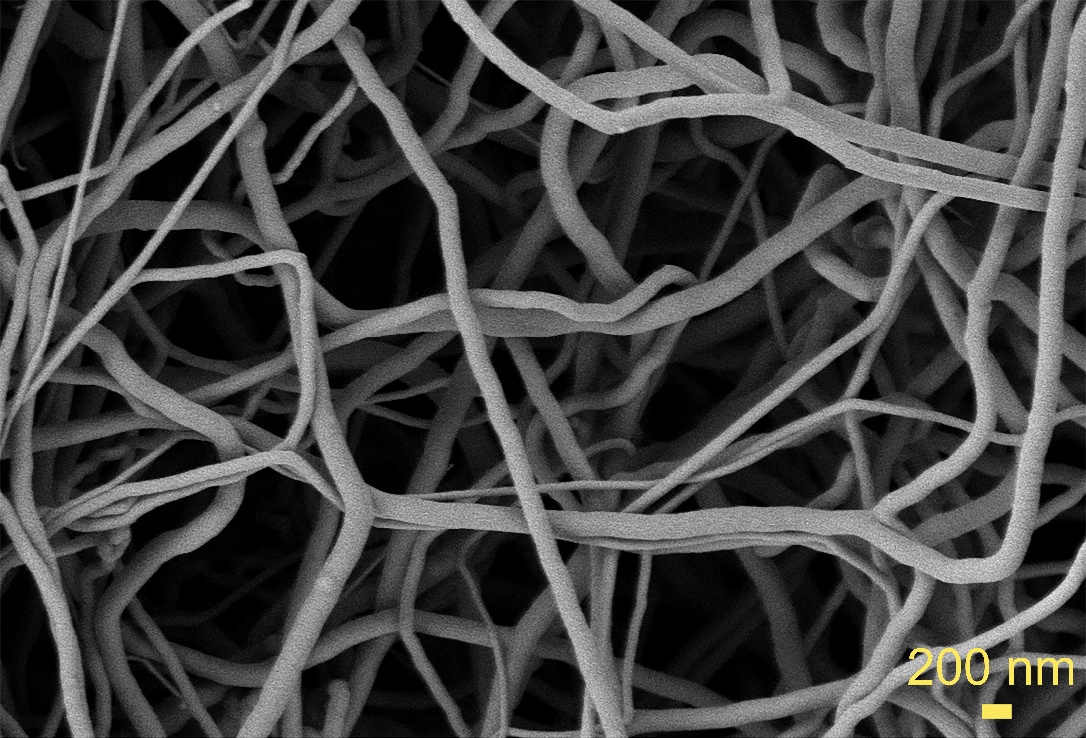


**Figure S3.** SEM image of the C-NWN nanostructure.


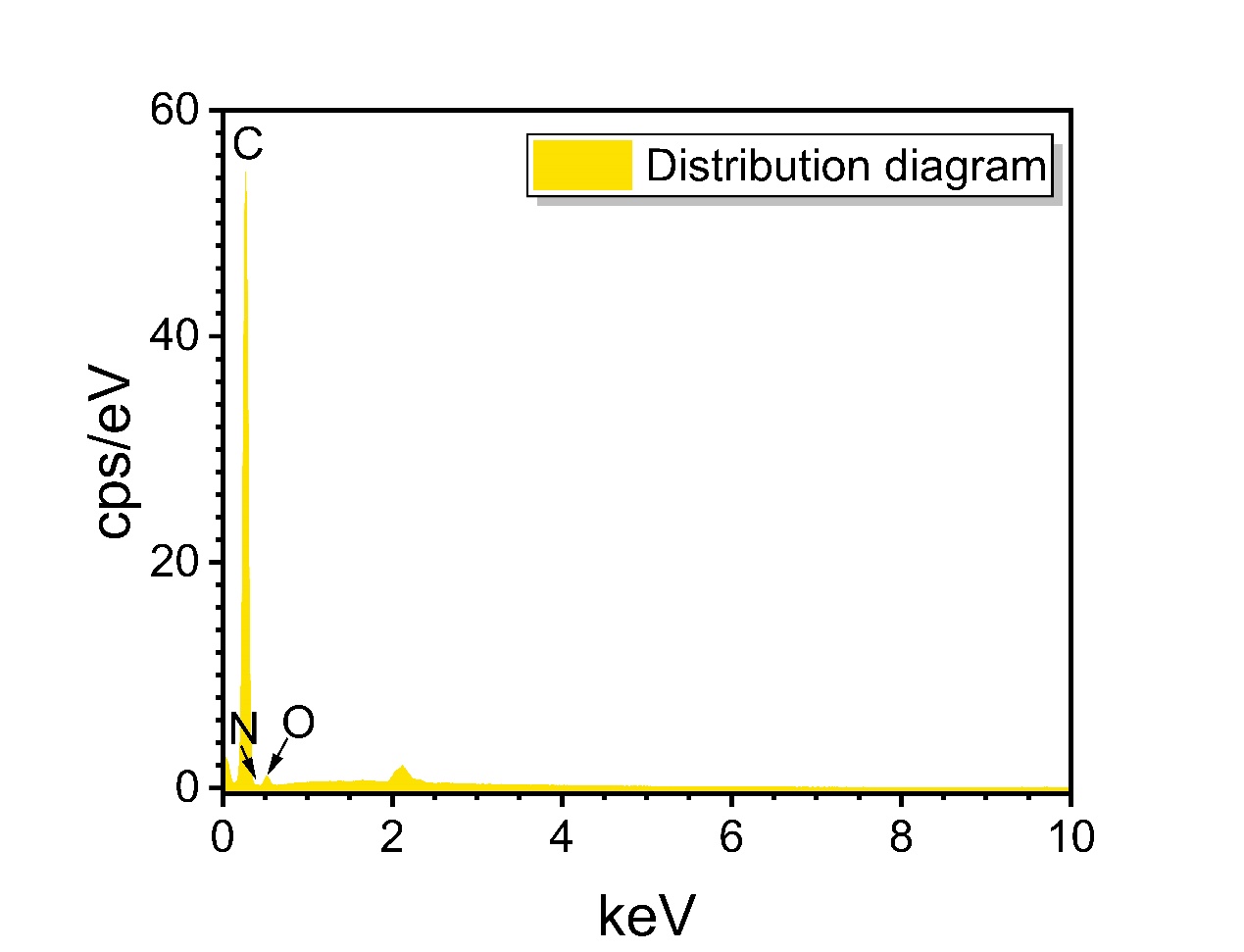


**Figure S4.** EDS distribution diagram of CC@C-NWN.


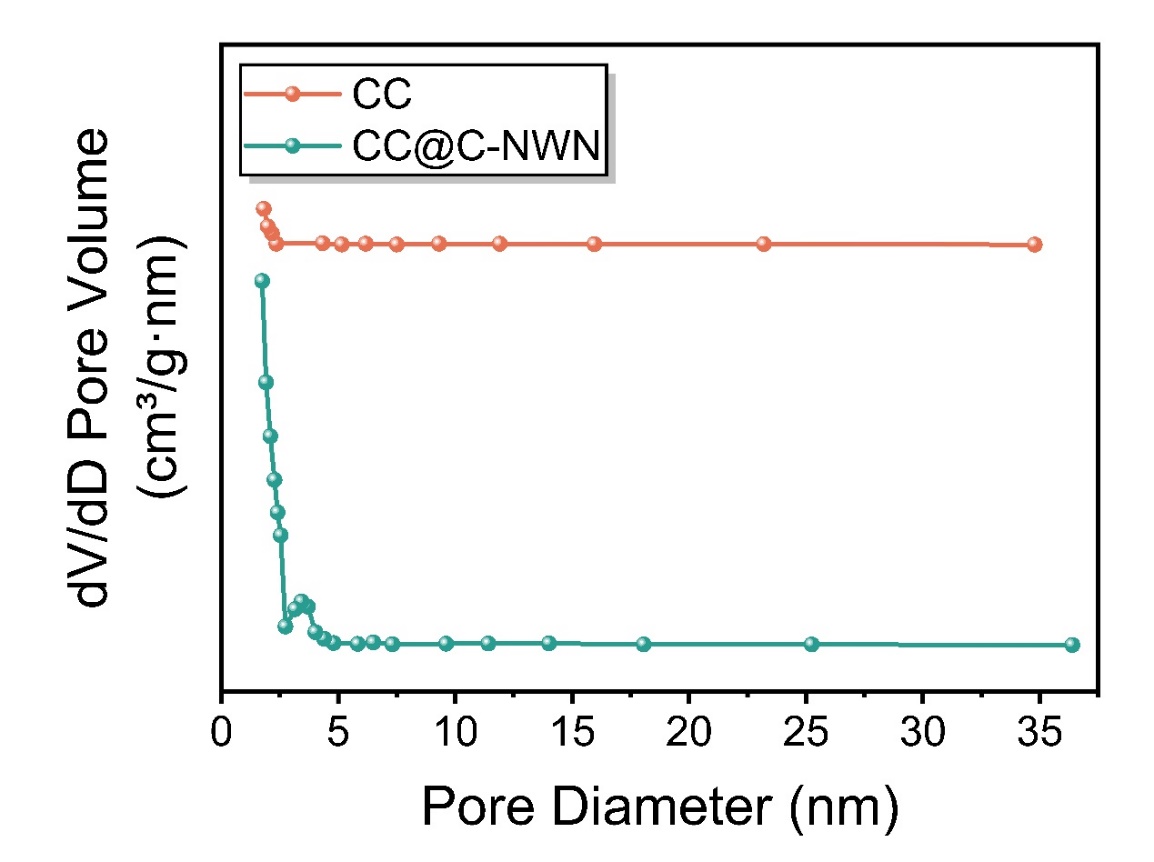


**Figure S5.** Pore size distribution of CC and CC@C-NWN.


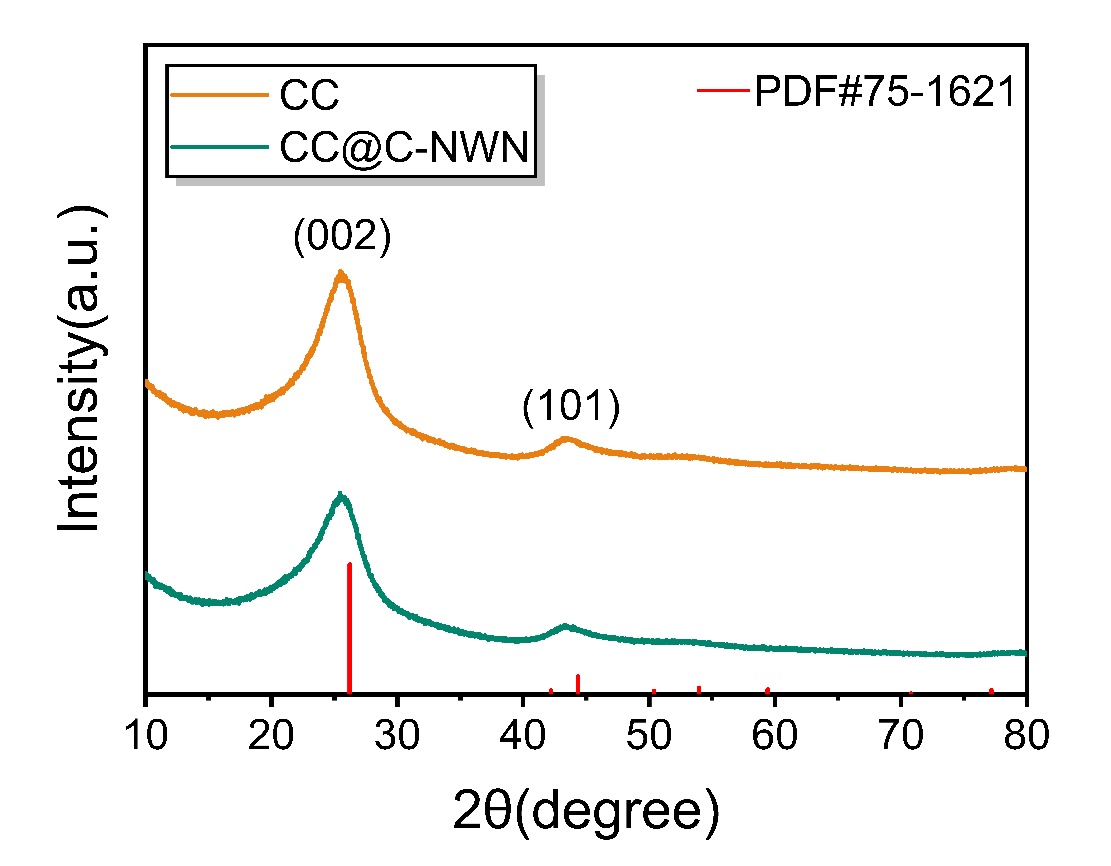


**Figure S6.** XRD pattern of CC and CC@C-NWN.


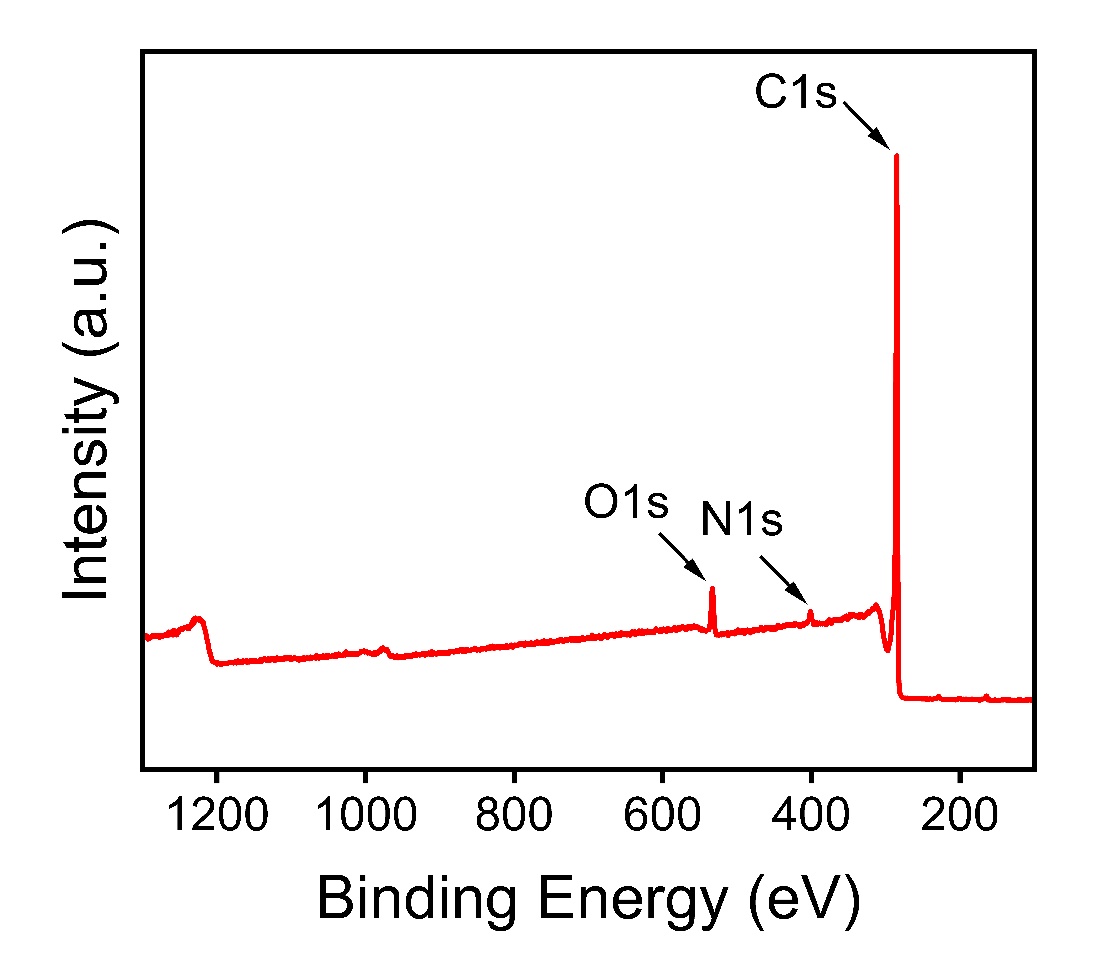


**Figure S7.** XPS survey of CC@C-NWN.


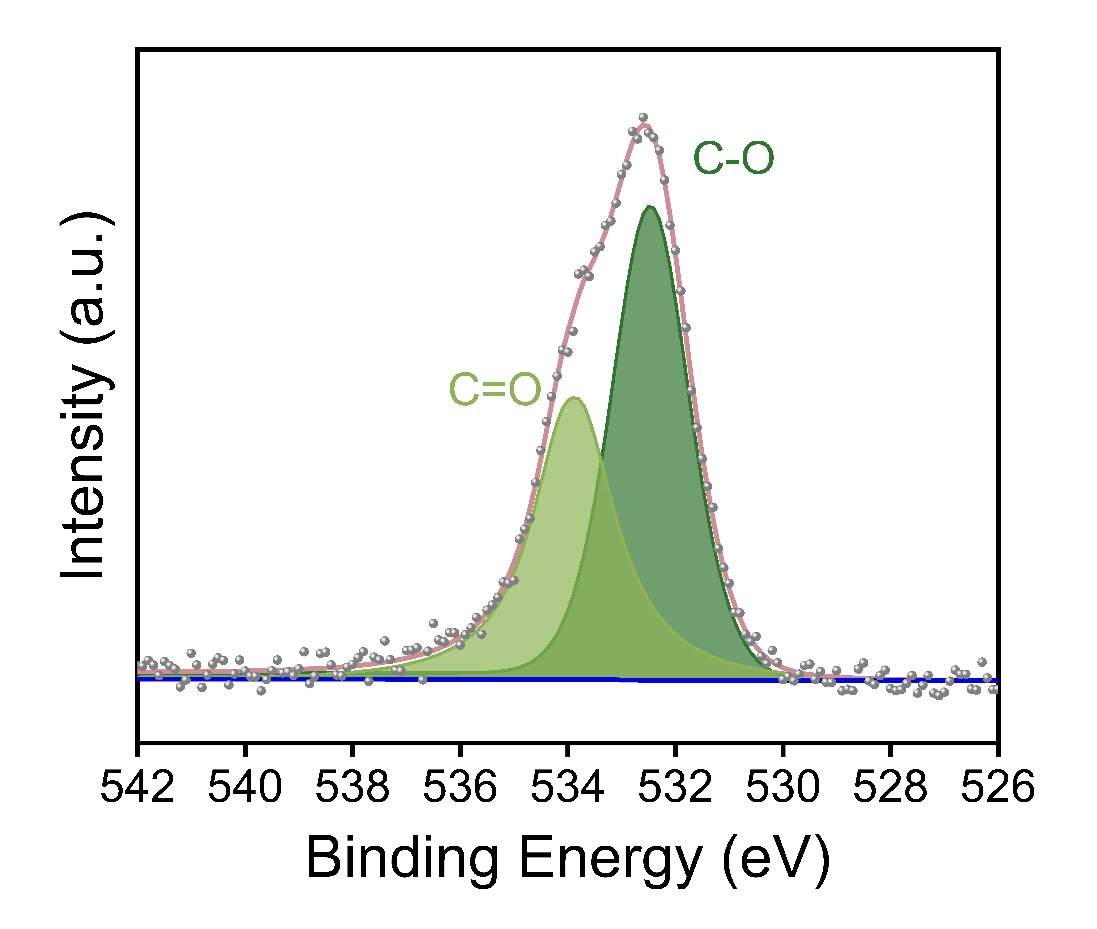


**Figure S8.** The O1s spectrum of CC.


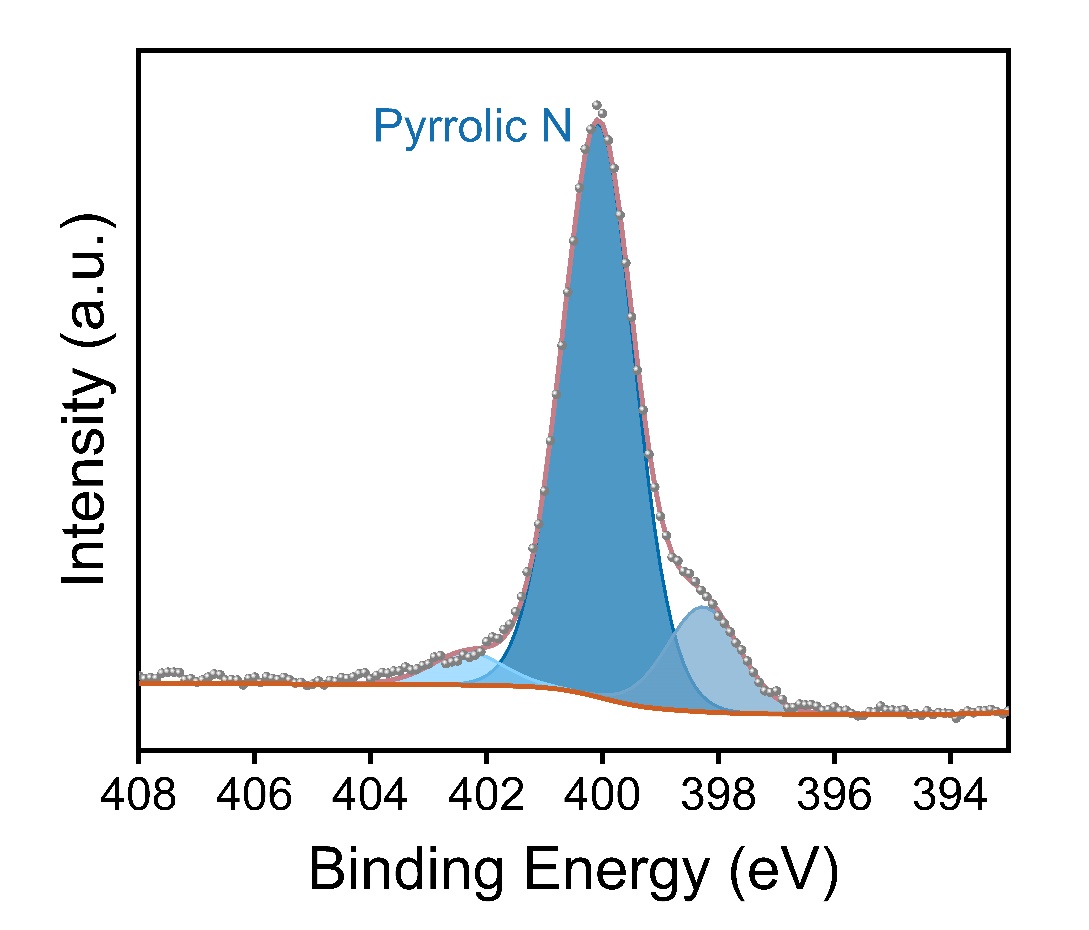


**Figure S9.** The N1s XPS spectrum of PPy-NWN@CC.


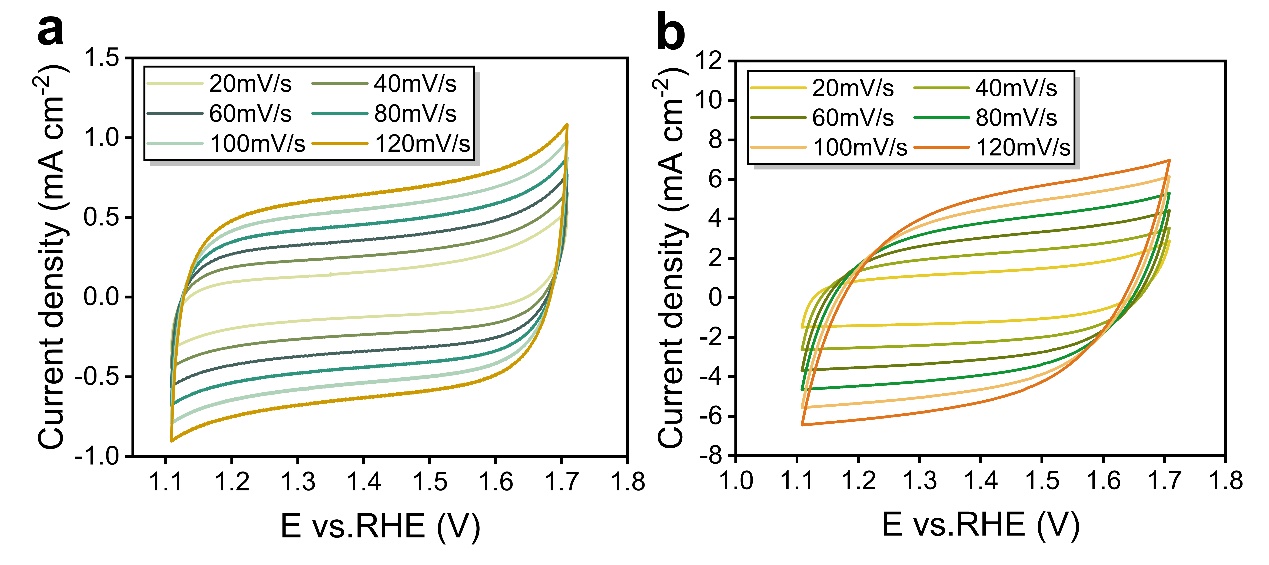


**Figure S10.** a) CV curves of CC at the scanning rates of 20–120 mV s^−1^. b) CV curves of CC@C-NWN at the scanning rates of 20–120 mV s^−1^.


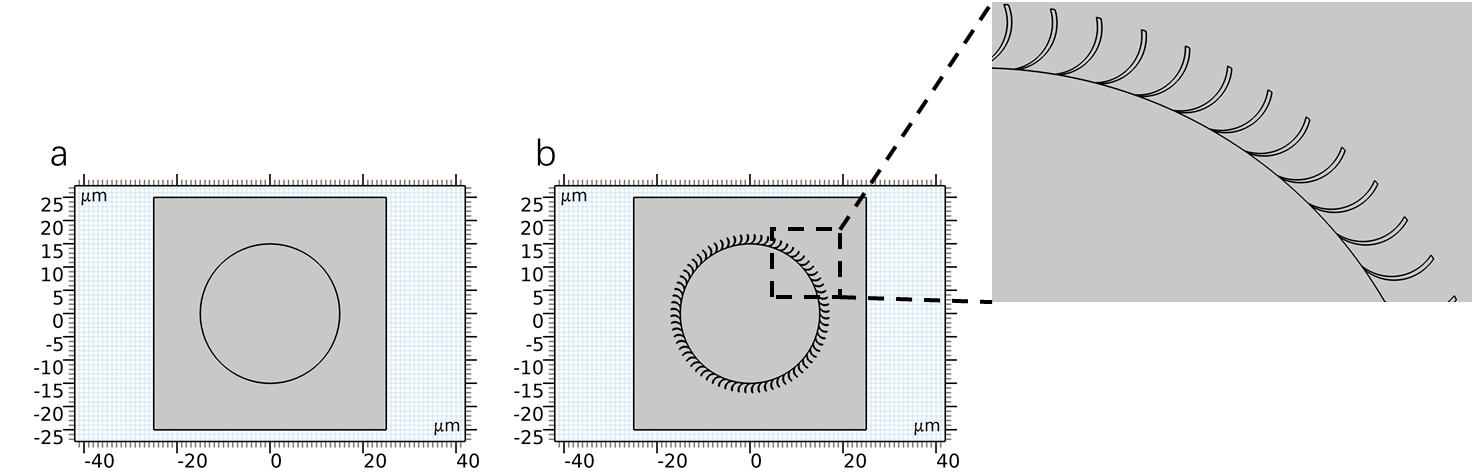


**Figure S11.** Two - dimensional geometric model of the fiber unit. a) CC. b) C@CC-NWN.


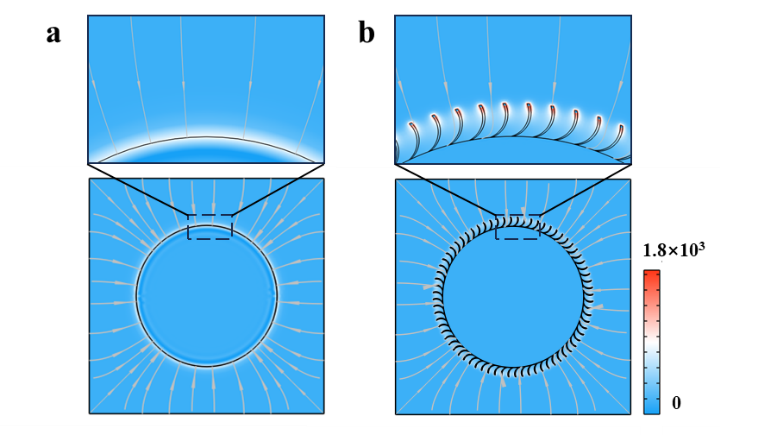


**Figure S12.** Effect of nanostructure on Cl⁻ concentration distribution. a) CC. b) CC@C-NWN.


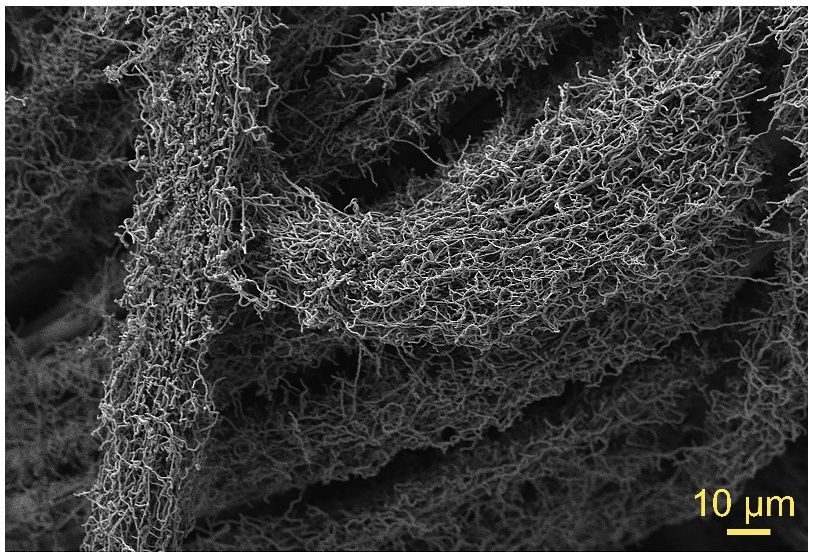


**Figure S13.** SEM images of the CC@C-NWN after 50 h chronopotentiometric test.


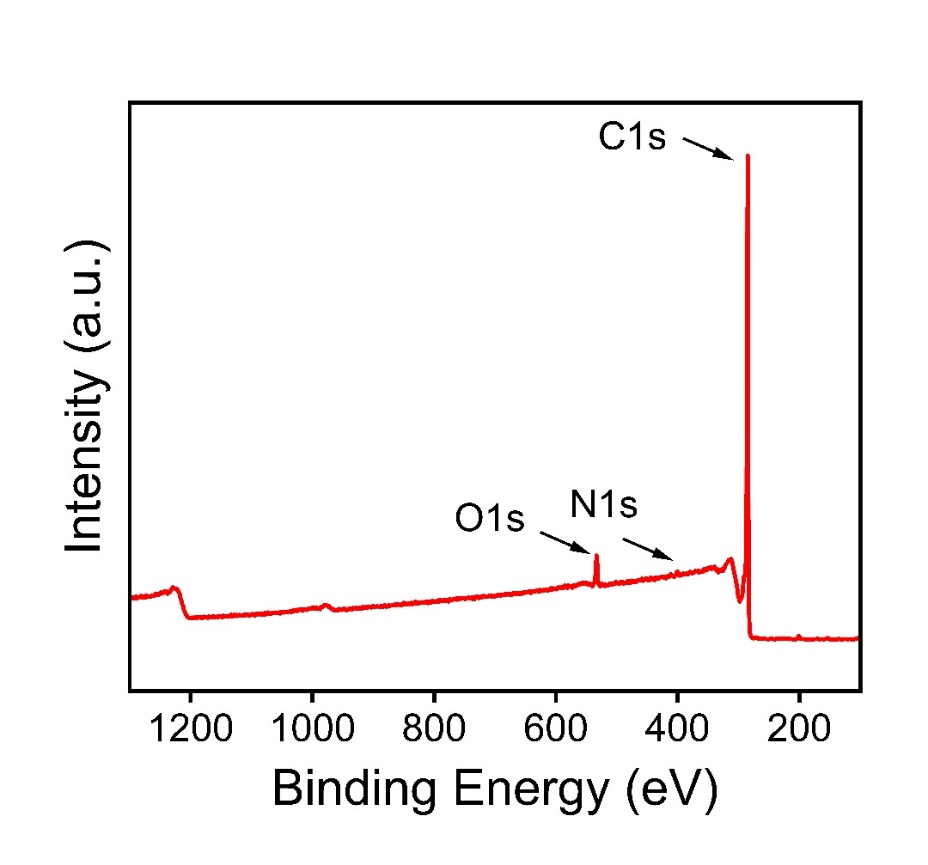


**Figure S14.** XPS survey of CC@C-NWN after 50 h chronopotentiometric test.


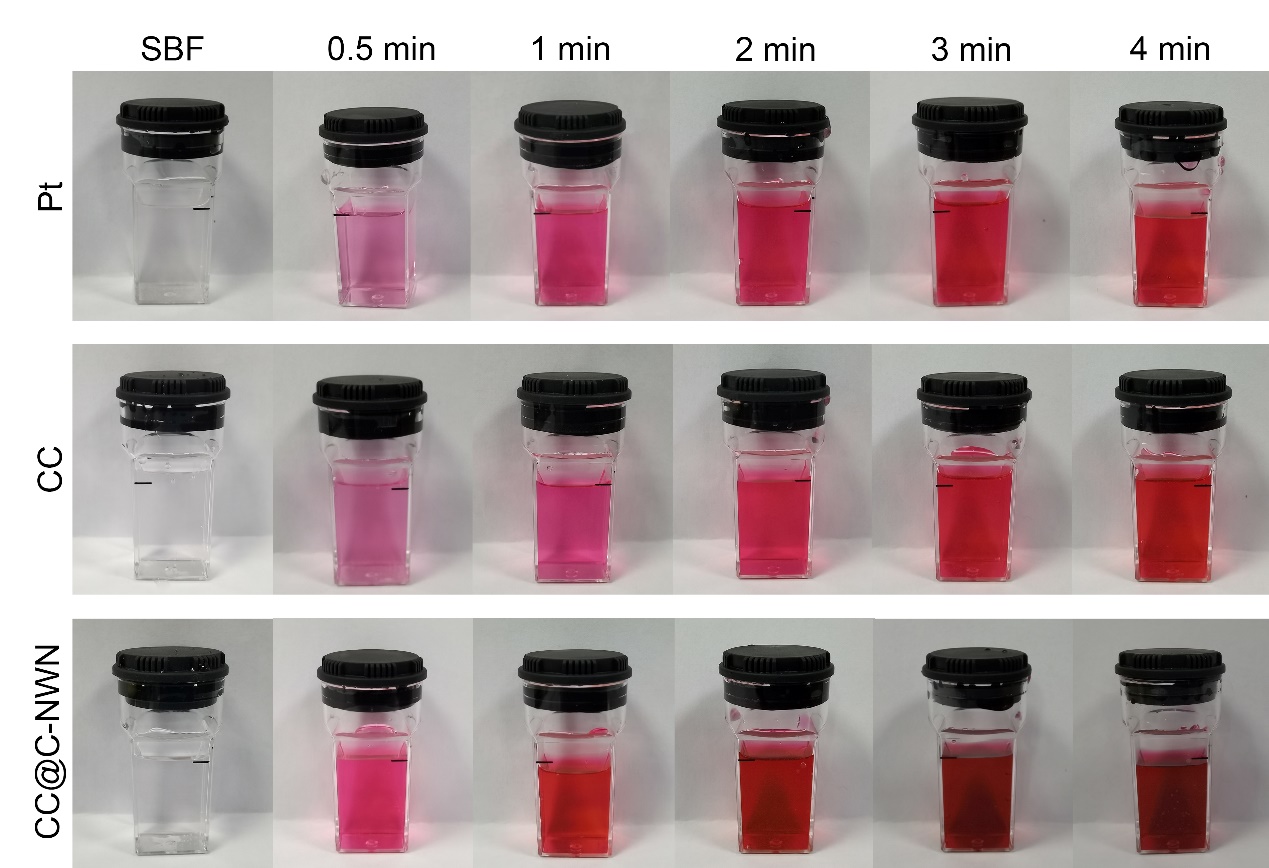


**Figure S15.** Under 3V DC condition, a comparative analysis of the coloration reaction of RCS produced by three types of electrode materials with the DPD reagent.


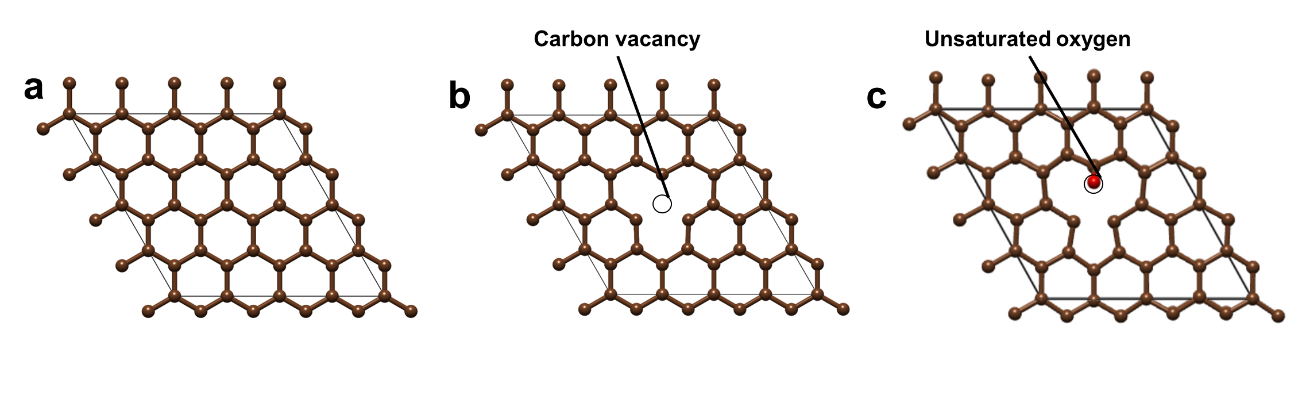


**Figure S16.** The models of a) pure carbon without vacancy (C), b) the carbon vacancy model (Cv), c) the unsaturated oxygen model (Co).


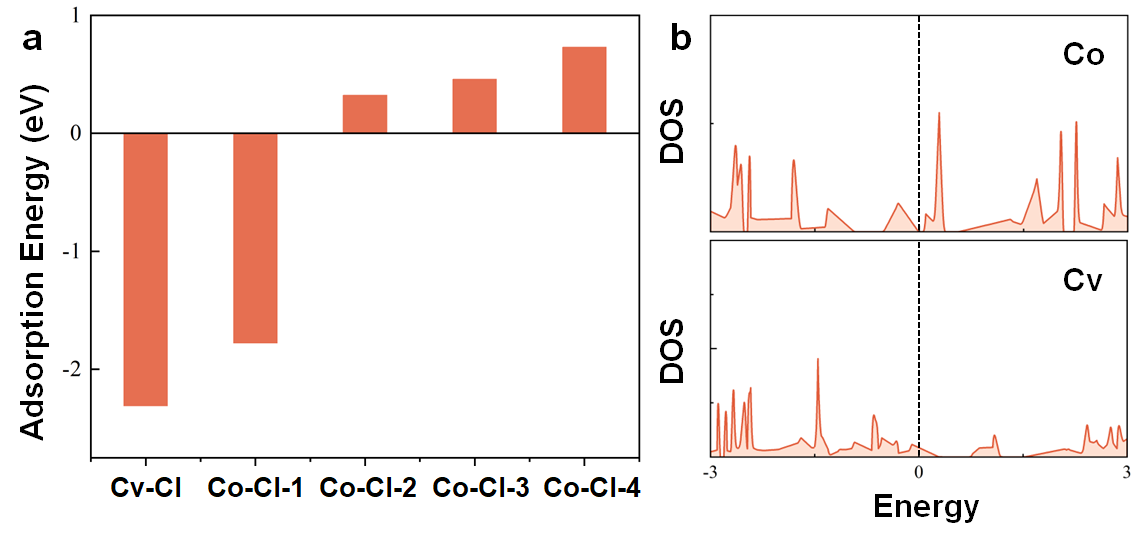


**Figure S17.** a) The adsorption energy of Cv and Co to Cl^-^. b) The DOS of Co and Cv.

**
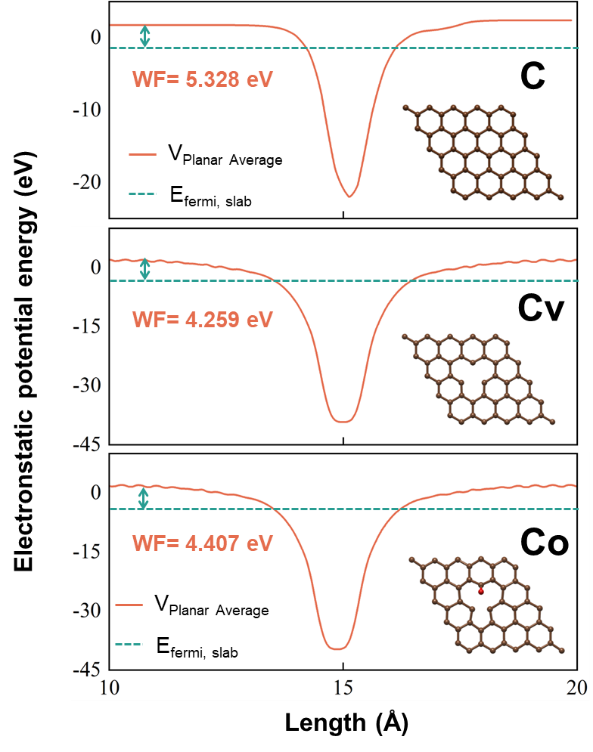
**

**Figure S18.** The work function of C, Cv and Co surface.


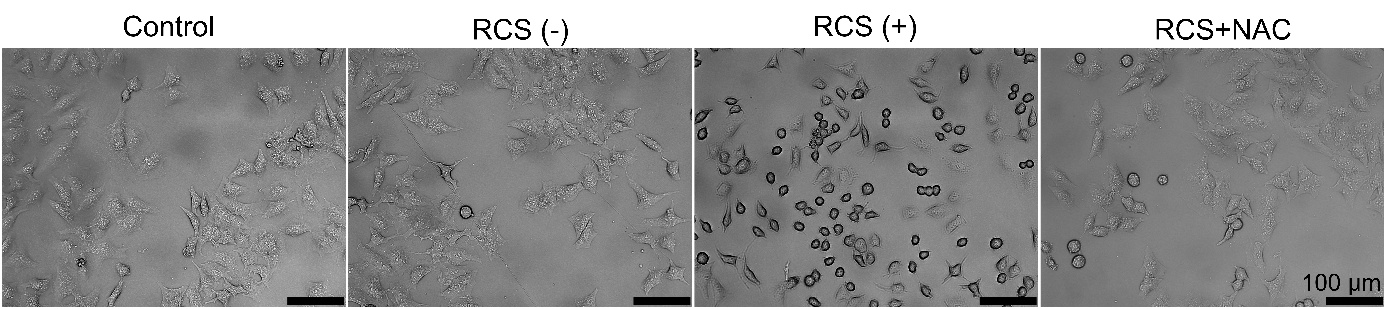


**Figure S19.** The bright-field image for intracellular RCS (Scale bar: 100 μm).


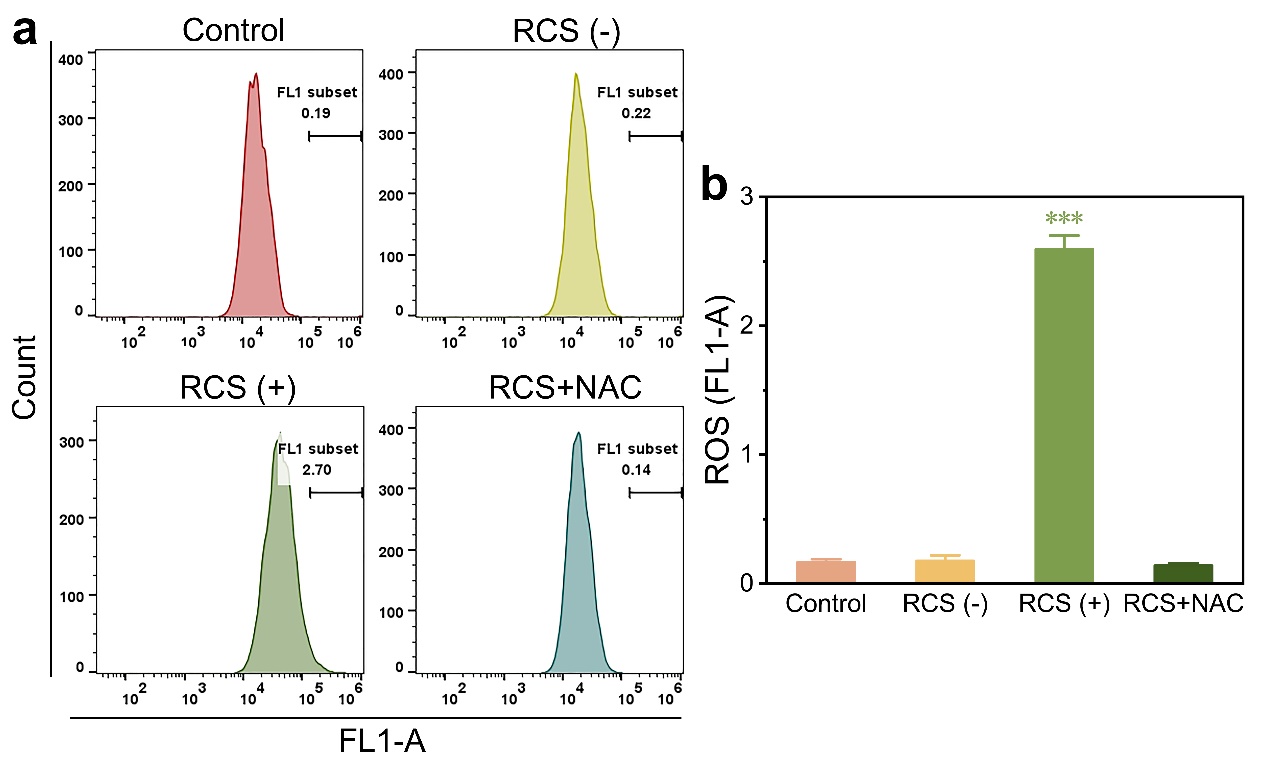


**Figure S20.** a) Flow cytometry diagram for intracellular RCS. b) Statistical graph of intracellular RCS fluorescence intensity (* p＜0.05, ** P＜0.01, *** P＜0.001).


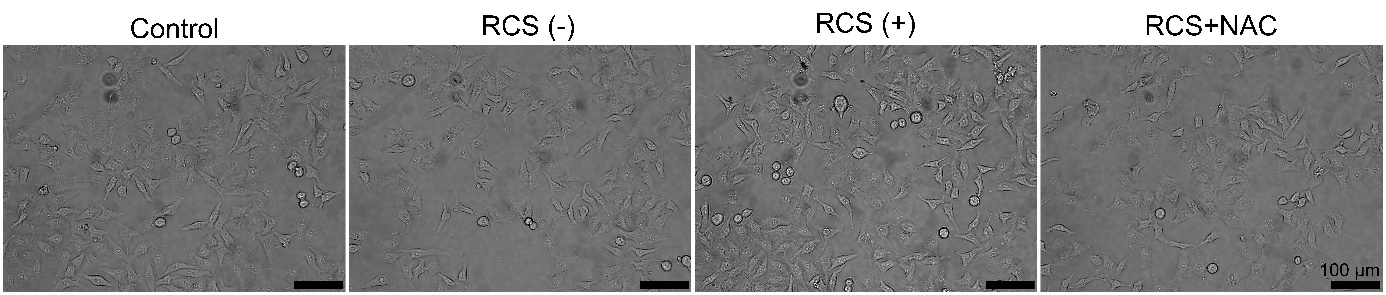


**Figure S21.** The bright-field image for JC-1 staining (Scale bar: 100 μm).


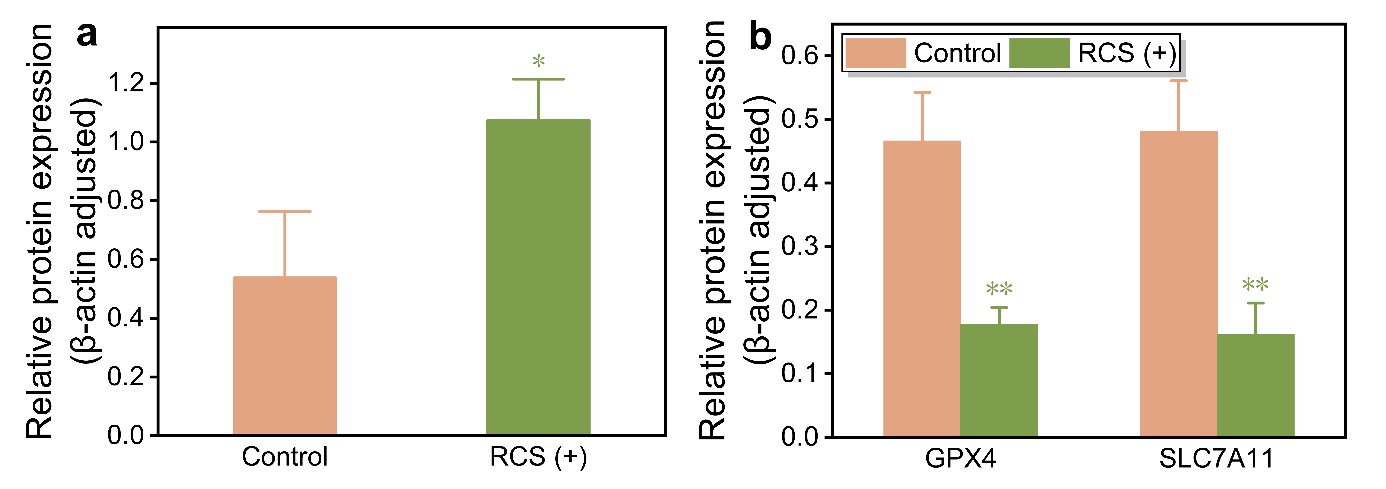


**Figure S22.** a) Statistical graph of c-caspase 3 expression level. b) Statistical graph of GPX4 and SLC7A11 3 expression levels (* p＜0.05, ** P＜0.01, *** P＜0.001).


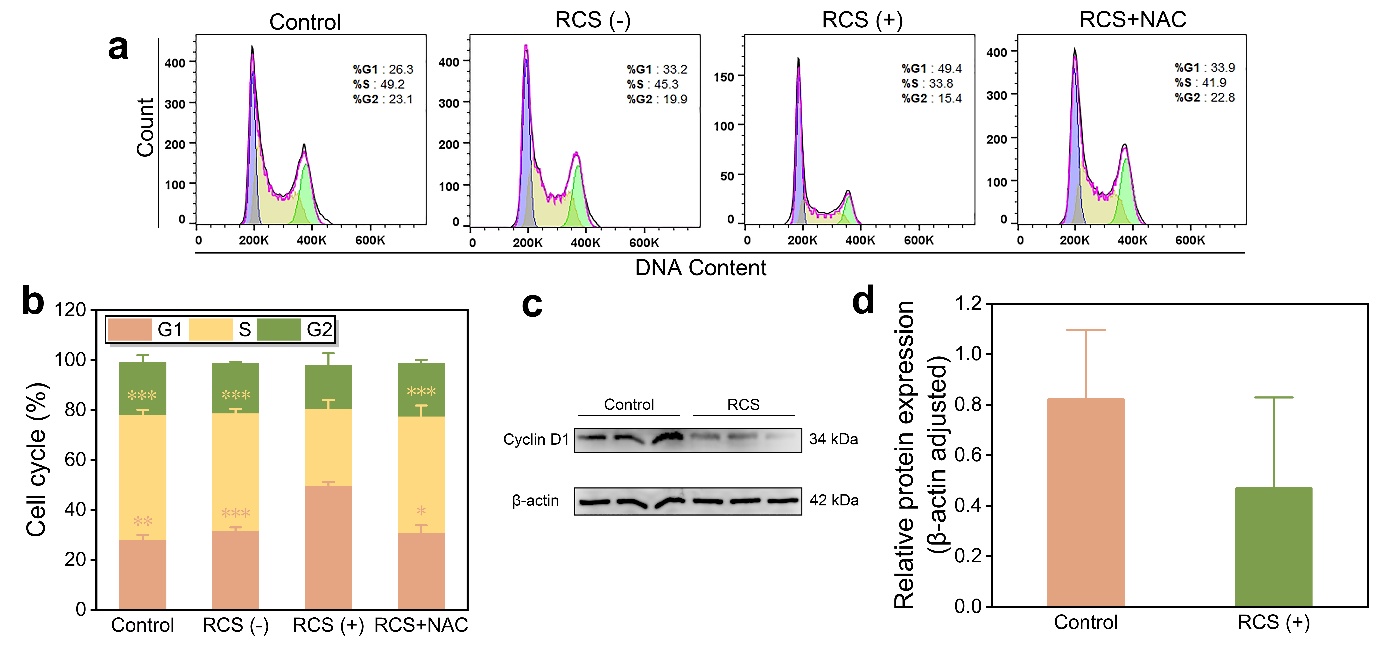


**Figure S23.** a) Cell cycle distribution measured by flow cytometry. b) Cell cycle distribution data. c-d) Expression of Cyclin D1 evaluated by Western blot analysis (* p＜0.05, ** P＜0.01, *** P＜0.001).


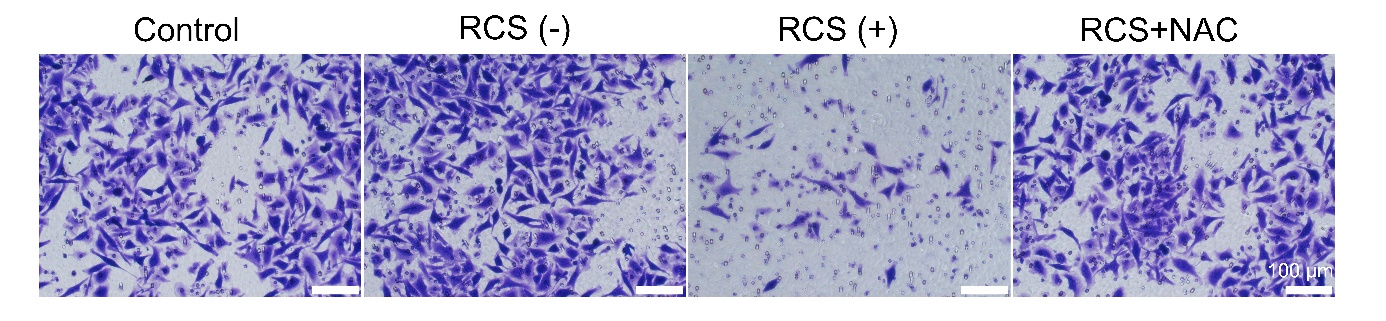


**Figure S24.** Microscopic images of crystal violet staining in Transwell assay (Scale bar: 100μm).


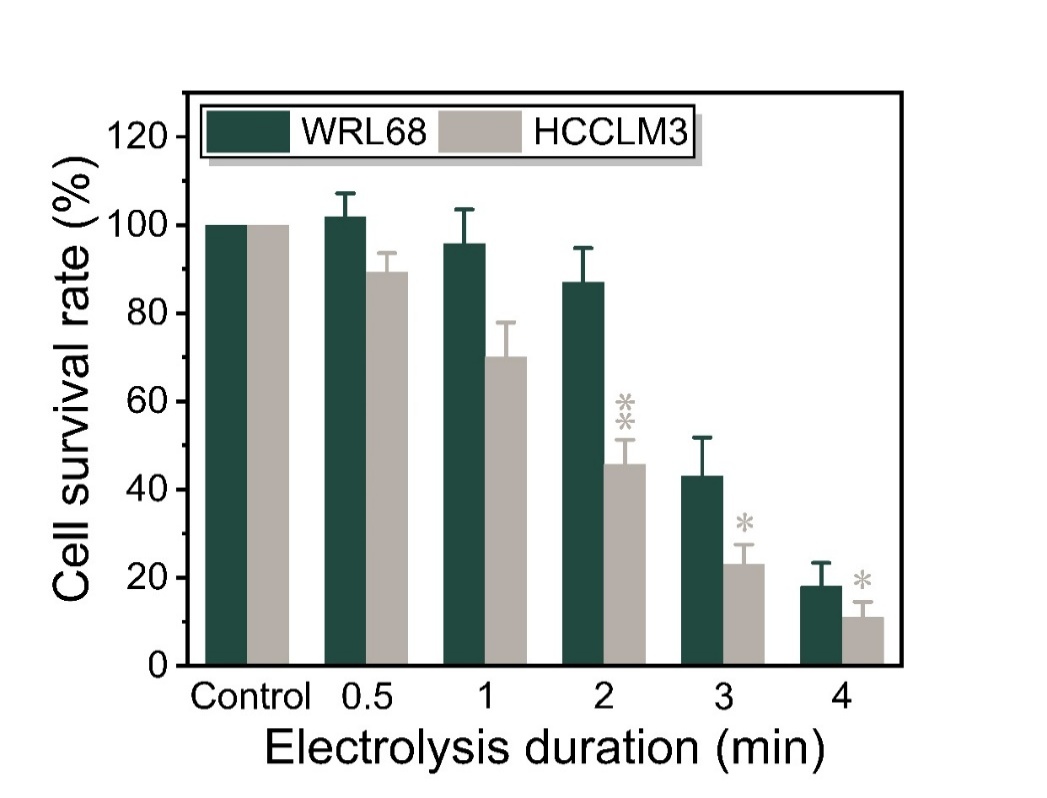


**Figure S25.** The comparison of cell survival rates between WRL68 and HCCLM3 after treatment with RCS generated from the CC@C-NWN electrochemical system under various electrolysis durations (* p＜0.05, ** P＜0.01, *** P＜0.001).


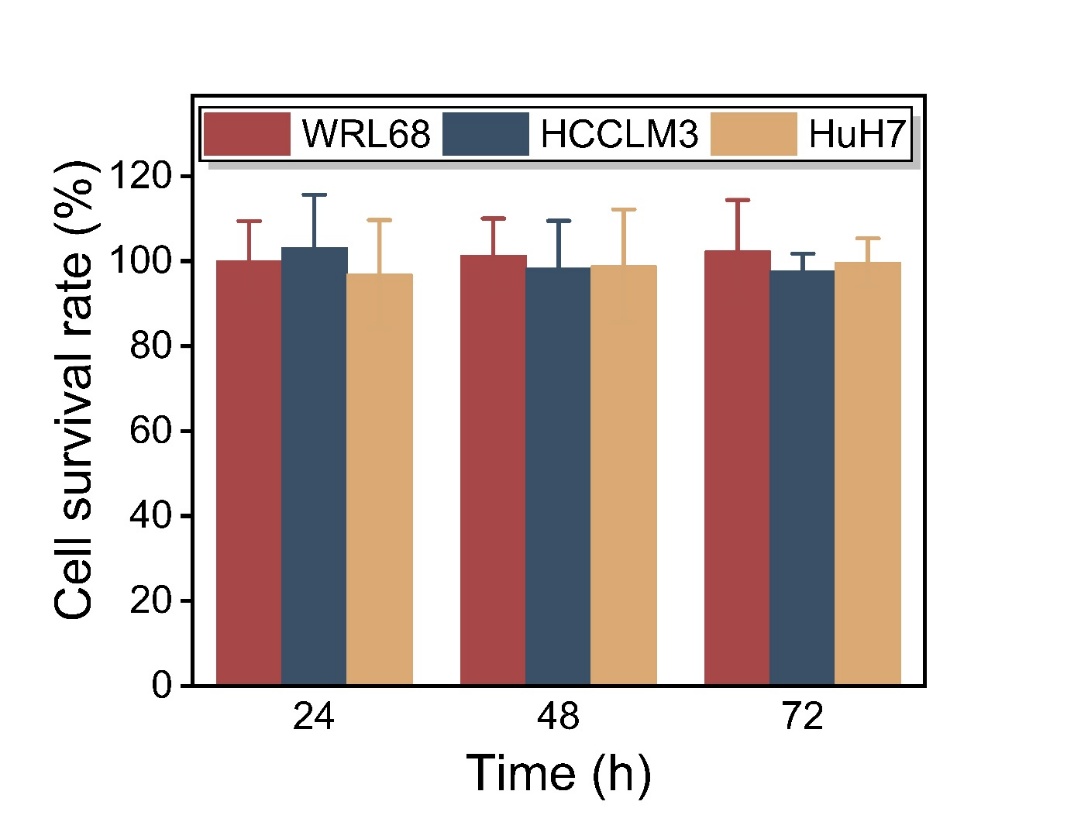


**Figure S26.** Cell survival rates of WRL68, HCCLM3 and HuH7 cells after being co-cultured with CC@C-NWN for 24, 48, and 72 h.


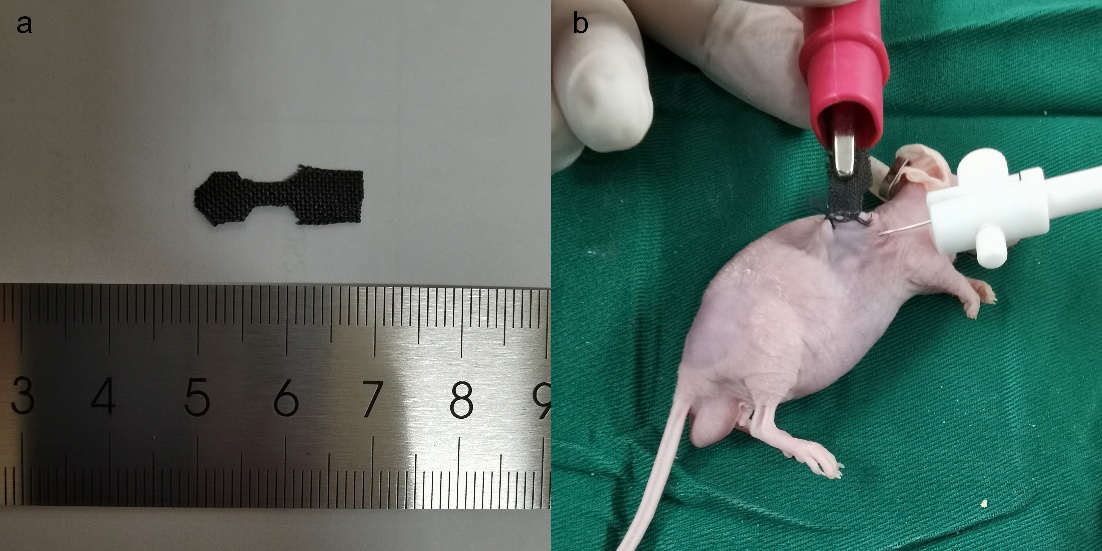


**Figure 27.** a) The cutting method of CC/CC@C-NWN. b) The electrotherapy modality for mice.


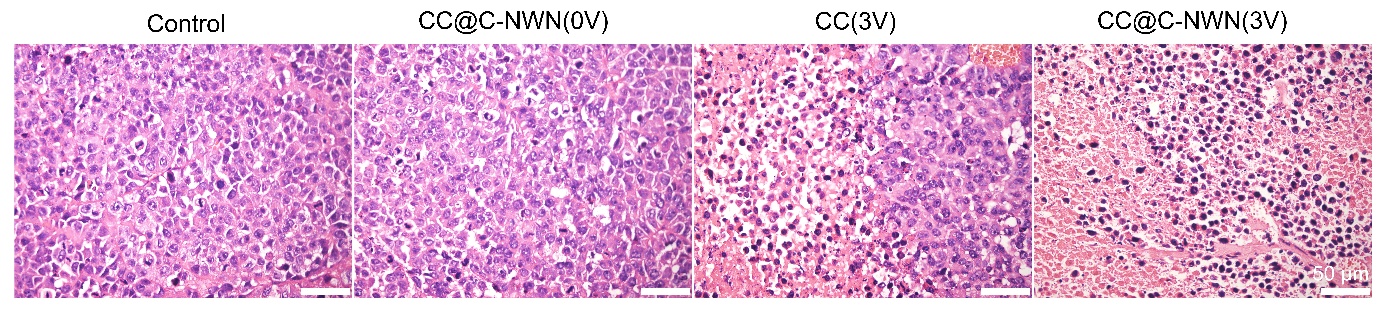


**Figure S28.** H&E staining of tumor tissues (Scale bar: 50 μm).


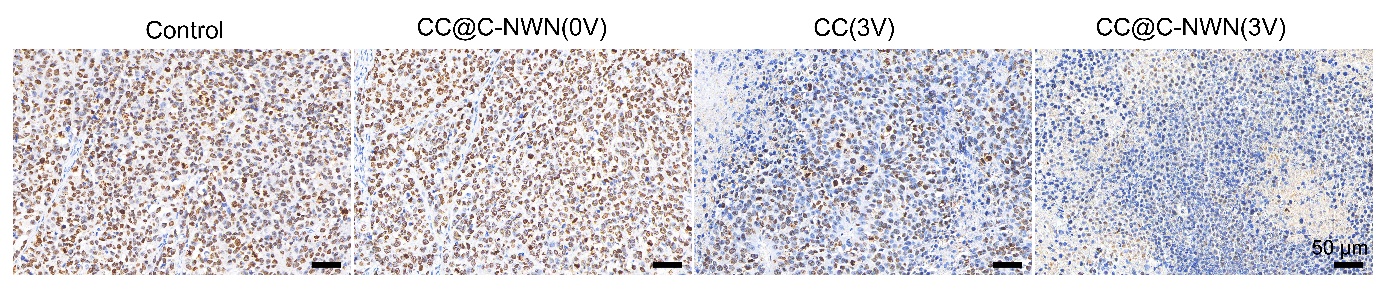


**Figure S29.** Ki-67 staining of tumor sections (Scale bar: 50 μm).


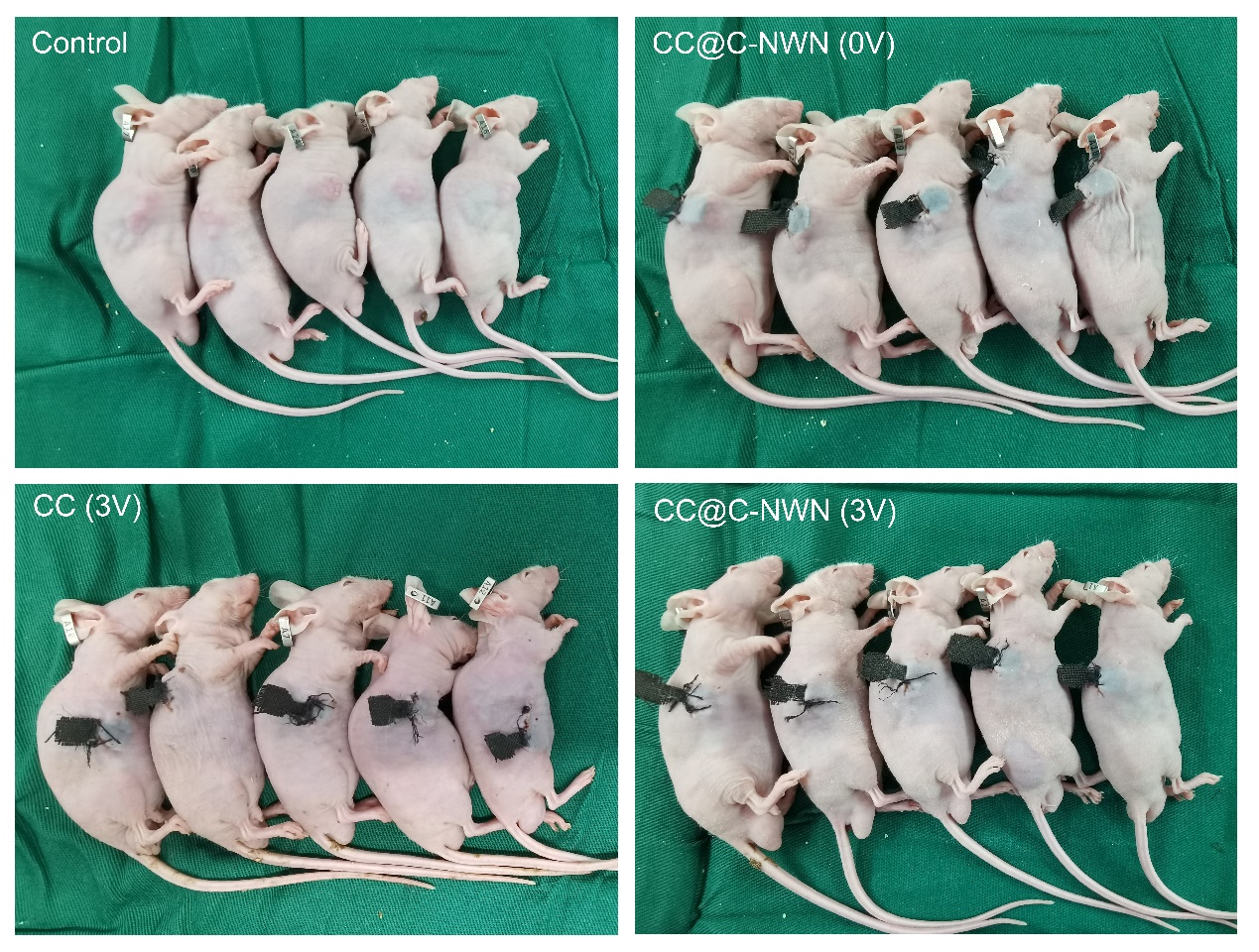


**Figure S30.** Photos of the mice following treatment completion.


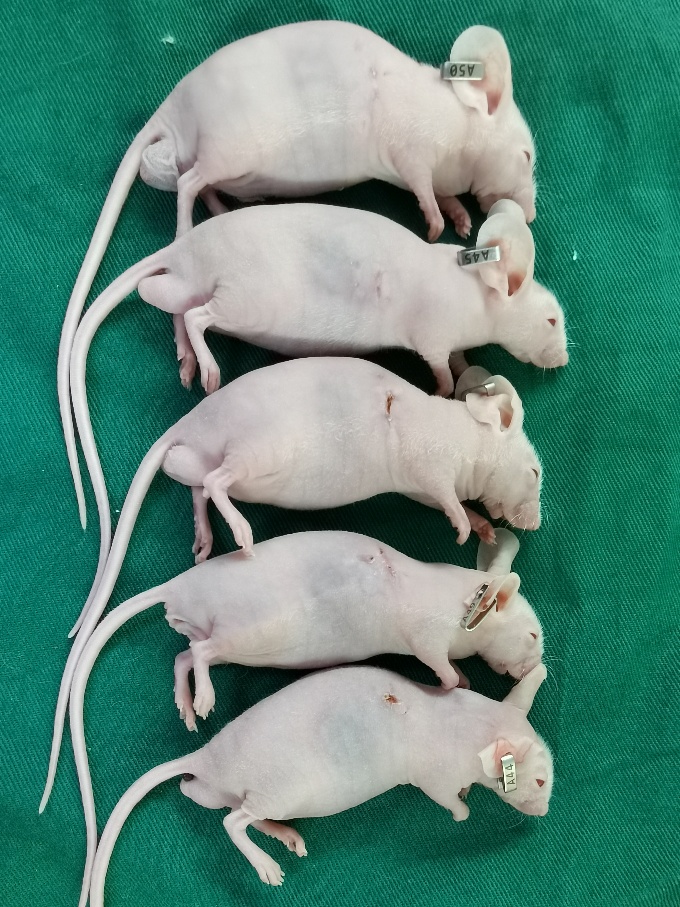


**Figure S31.** The healing condition of the skin in CC@C-NWN (3V) group mice following suture closure.


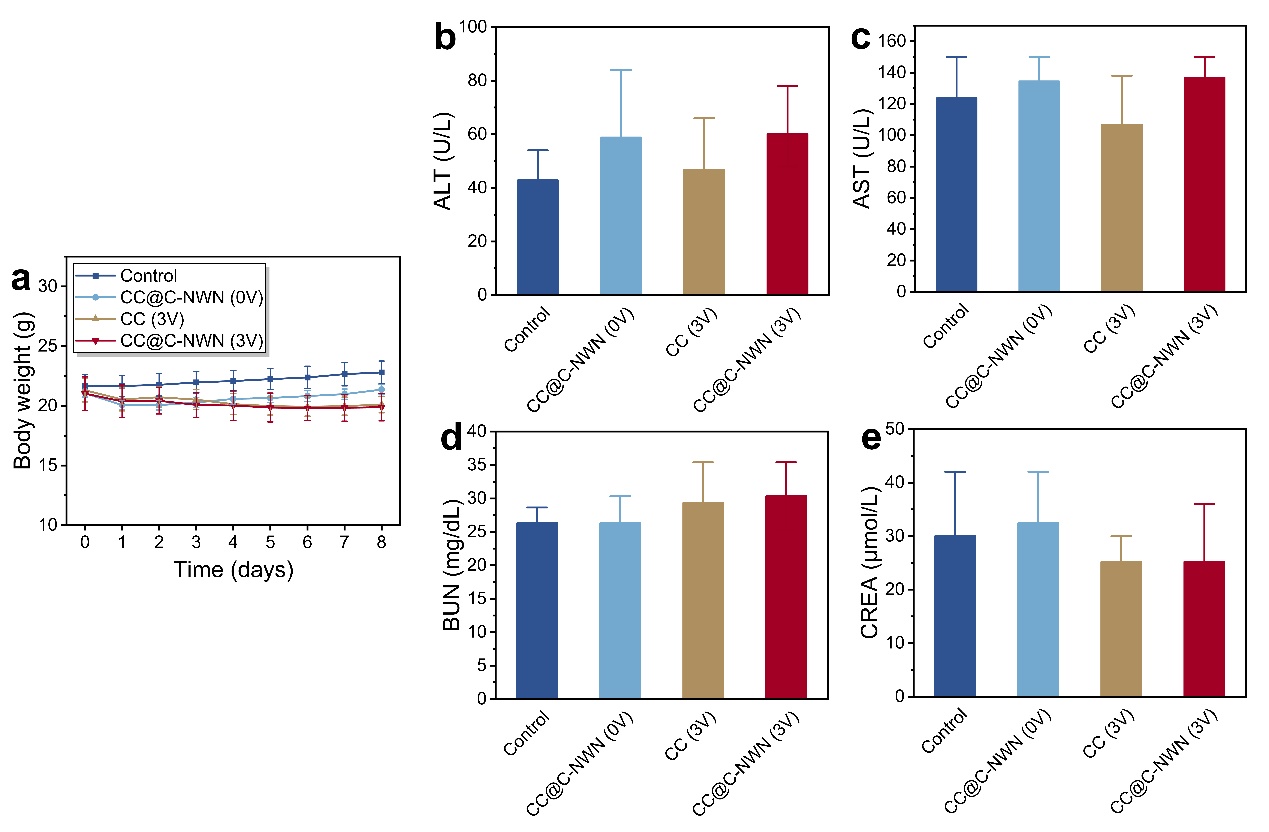


**Figure S32.** a) Body weight changes in mice across different groups. At the end of the treatment (day 8), the levels of b) ALT, c) AST, d) BUN, and e) CREA in mice.


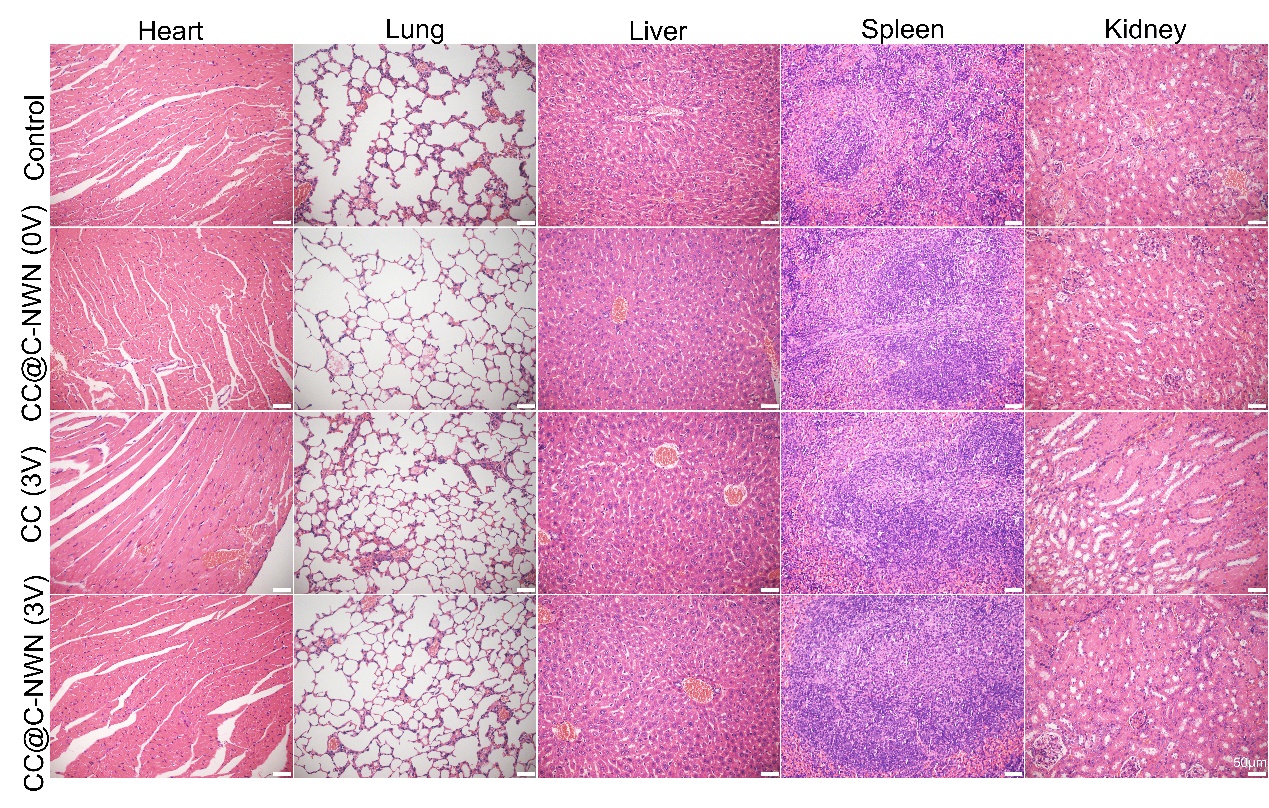


**Figure S33.** H&E staining sections from major organs of mice. (scale bar: 50 μm)


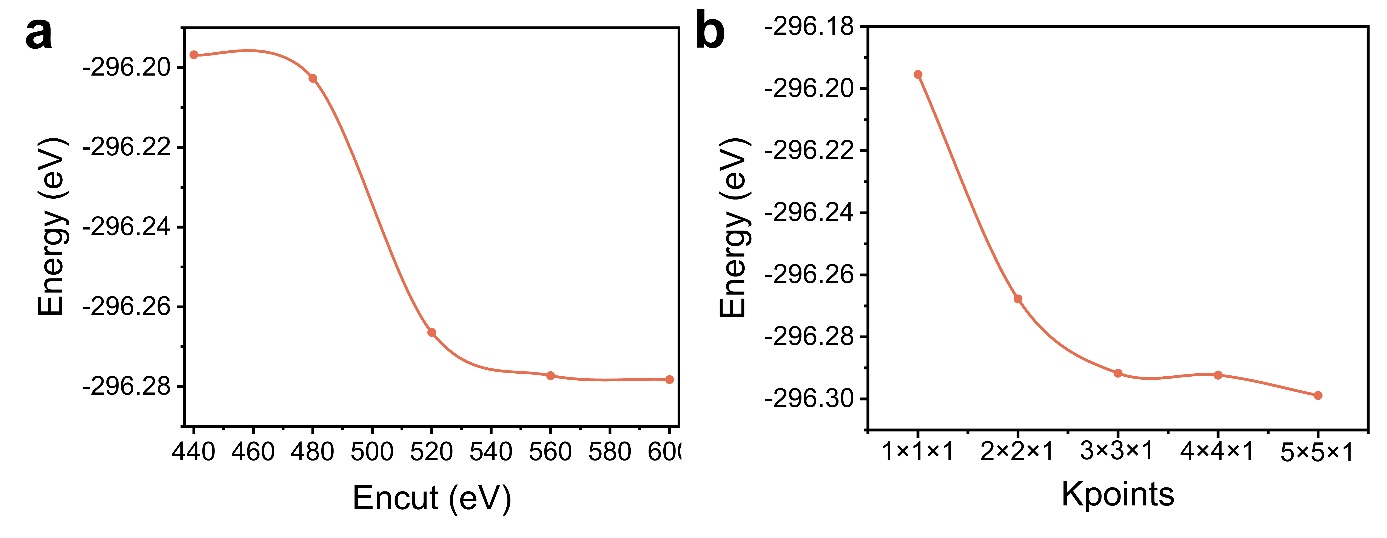


**Figure S34.** The convergence tests of a) Encut and b) Kpoints.
